# Identifying and ranking drivers of green manufacturing for sustainable industrial practices

**DOI:** 10.1038/s41598-026-44849-y

**Published:** 2026-04-08

**Authors:** Shivakar Prasad, Rakesh Kumar, Satish Namdev

**Affiliations:** 1 Department of Mechanical Engineering, Manipal University Jaipur, Jaipur, 303007 Rajasthan India; 2 Automobile Engineering, Government Polytechnic, Aadampur, Tarabganj, Gonda, 271401 Uttar Pradesh India

**Keywords:** Green manufacturing, Sustainable manufacturing, Drivers, Sustainability Random Forest regression, Indian industry, Environmental strategy, Business and management, Business and management, Economics, Economics, Environmental social sciences, Environmental studies, Operational research

## Abstract

This research investigates the critical factors that influence the adoption and effectiveness of green manufacturing in Indian industries. Drawing on survey data from 100 firms across Uttar Pradesh, twelve key drivers were identified and evaluated to understand their relative importance in shaping sustainable practices. A total of 100 firms were approached, out of which 45 complete responses were used for random forest modelling. The study highlights the central role of economic benefits, business reputation, regulatory compliance, and stakeholder engagement in advancing green initiatives. Validation with industry experts confirmed the practical relevance of these findings, which align with measurable improvements in energy efficiency, waste reduction, and production optimization. The analysis demonstrates that firms prioritizing high-impact drivers achieve notable gains in both environmental performance and competitiveness. While the study is region-specific, the framework is adaptable to other sectors and geographies. The results provide managers and policymakers with actionable insights to allocate resources more effectively and design strategies that accelerate the transition toward sustainable manufacturing systems. This work bridges the gap between theoretical models and industrial application, contributing to both academic discourse and real-world implementation of environmentally responsible production.

**Supplementary Information:**

The online version contains supplementary material available at 10.1038/s41598-026-44849-y.

## Introduction

Manufacturing has been essential to global marketplace since industrial revolution, providing jobs, conveniences, and economies^1^. This tendency is expected to endure as world’s rising population demand larger quantities of products^2,3^. Growing industrial activity has intensified environmental challenges, increasing need for cleaner production^4–7^. To address this issue, green manufacturing was established to modify production processes. Manufacturing industries are reconsidering their processes to produce greener products and services^8–10^.

Research suggests that green alternatives or green manufacturing practices are a viable solution for industries that are vulnerable^11^. Green manufacturing uses cleaner processes to reduce resource use, waste, and emissions while improving efficiency^12–15^. This method encourages eco-efficiency through waste reduction and resource optimization, leading to improved productivity and cost savings, which ultimately influences return on investment^16–18^. For example, minimizing energy usage and material waste directly decreases production expenses while enhancing system efficiency. Moreover, with the increasing consumer awareness regarding environmental concerns, sustainable manufacturing presents avenues for enhancing market presence by attracting environmentally aware customers and elevating product quality^19^. In contrast to traditional approaches that typically prioritize either cost or time efficiency, sustainable practices enhance all aspects of competitive manufacturing, including cost, time, flexibility, and quality^20^. Across the globe, there is a growing trend among governments and regulatory bodies to enforce green manufacturing practices through a combination of incentives, penalties, and compliance mandates, transforming it from an option into an obligation Hong Kong Green Manufacturing Alliance Report, 2008). These initiatives illustrate a worldwide effort aimed at sustainability, characterized by fulfilling current requirements without jeopardizing the needs of future generations^21^. Through the reduction of hazardous emissions, the elimination of wasteful resource consumption, and the integration of recycling practices, green manufacturing promotes environmental preservation while upholding economic and social goals. This framework highlights the importance of sustainability as a foundational concept and green manufacturing as its applicable approach. The implementation of green manufacturing is supported not only by environmental considerations but also by its economic and regulatory benefits, forming a holistic approach to competitive and sustainable business practices^22^. The combination of green technology and a strategic approach enhance its significance, establishing it as an essential element of contemporary industrial change.

The initiative launched by the Indian government seeks to establish India as a prominent player in the global manufacturing sector by promoting investment and embracing cutting-edge manufacturing techniques^23^. The initiative focuses on creating products and services that are flawless to reduce returns, while also committing to eco-friendly practices to promote sustainability^24^. This highlights the government’s aim to incorporate sustainability within the manufacturing sector, viewing green manufacturing as an essential element for national industrial and economic success^25^. Green manufacturing integrates sustainable practices through the application of advanced technologies and processes that match or exceed the productivity and efficiency of conventional methods while being more environmentally friendly^26^. Although previous studies recognize the importance of green manufacturing in Indian manufacturing, there is a lack of systematic identification and ranking of its enablers and barriers^27^. A significant portion of current studies offers a broad overview of green manufacturing, frequently limited to specific regions or focused on literary critiques, failing to consider the practical aspects of implementation^28^. Furthermore, investigations into the factors influencing green manufacturing have inadequately addressed the diverse environmental and social elements present in India’s multifaceted cultural and industrial contexts^29^. This highlights a disconnect between theoretical frameworks and practical implementation, underscoring the need for thorough, evidence-based strategies to identify, evaluate, and tackle green manufacturing facilitators and obstacles in the Indian manufacturing landscape^30^. The present study is guided by five important research questions that shape overall investigation. These include:

(i) What are essential drivers that influence adoption of green manufacturing in Indian manufacturing industries?
(ii) How effectively can Random Forest Regression be applied to identify, quantify, and prioritize these twelve drivers using existing industrial data?
(iii) Which drivers exhibit highest relative importance in predicting green manufacturing success and thereby demand greater managerial focus?
(iv) To what extent does the proposed dual-phase framework, combining literature review, expert judgment, and industrial validation, bridge gap between theoretical models and practical application?

These research questions collectively guide the methodological approach and ensure that the study contributes both theoretical insight and practical value to sustainable manufacturing.

## Literature review

### State of art for green manufacturing

Green manufacturing has garnered significant attention in recent decades as a strategic approach to integrate environmental sustainability into production processes. Early efforts in green manufacturing, such as the introduction of British Standard 7750 in the 1990s and the ISO 14001 Environmental Management System standard in 1996, Morrow and Rondinelli^31^. Sarkis and Rasheed (1995) highlighted that environmentalism had evolved from regulatory compliance to a strategic advantage, emphasizing energy conservation, pollution prevention, and ecological protection. They argued that environmentally conscious manufacturing focused on minimizing waste, designing recyclable products, and integrating sustainability into production strategies^32^. Noci et al. (1996) emphasized that growing environmental concerns compelled firms to integrate sustainability into supplier relationships and proposed a conceptual framework for evaluating suppliers’ environmental performance^33^. Handfield et al. (1997) examined the furniture industry and highlighted that integrating environmentally friendly practices across the entire value chain was essential for sustainable competitiveness. They emphasized that proactive environmental strategies needed to go beyond regulatory compliance by embedding green practices in design, procurement, manufacturing, and distribution^34^. Chin et al. (1999) examined the critical success factors for implementing ISO 14001-based EMS in Hong Kong manufacturing industries and highlighted the role of AHP in formulating effective strategies. Their study emphasized that adopting EMS enhanced environmental performance and supported global competitiveness despite associated costs^35^. Hui et al. (2001) reported that organizations demonstrated a positive attitude toward implementation, emphasizing its role in enhancing competitiveness^36^. Pun et al. (2002) emphasized that growing awareness of sustainable development encouraged, and they proposed a five-stage EMS planning framework that integrated strategic and environmental perspectives for guiding organizations in implementing sustainable management practices^37^. Matthews et al. (2003) examined the role of environmental benchmarking and found that while such systems organized environmental impacts and followed traditional benchmarking cycles, they lacked critical features needed for effective benchmarking. The study suggested extending these systems through consistent goal setting, centralized data reporting, and management review to enable knowledge transfer across facilities^38^. Gutowski et al. (2005) highlighted that environmentally benign manufacturing practices emerged as a competitive dimension across Japan, Europe, and the United States. They observed regional differences in approaches, with Japanese firms leveraging environmental advantages, European companies adopting protectionist policies, and U.S. firms showing awareness but lacking cohesive national strategies^39^. Sangwan et al. (2006) discussed the justification of green manufacturing systems through a performance value analysis model. They highlighted that such systems provided significant intangible benefits and competitive advantages compared to traditional approaches^40^. Vachon et al. (2007) examined the relationship between green supply chain practices and the adoption of environmental technologies, emphasizing supplier collaboration as a key driver of pollution prevention investments. They highlighted that customer collaboration had limited influence on environmental investment decisions within supply chains^41^. Li et al. (2010) proposed a methodology for selecting a green technology portfolio that considered both environmental strategies and synergies among technologies. Their work highlighted how enterprises could effectively integrate economic and environmental benefits into green manufacturing practices^42^. Lun et al. (2011) examined green management practices in container terminal operations and reported that environmentally friendly operations, supply chain cooperation, and internal management support enhanced firm performance. Their study demonstrated that the adoption of green management practices positively influenced organizational efficiency and competitiveness^43^. Searcy et al. (2012) highlighted the challenges of implementing ISO 14001, emphasizing synergies among system elements, auditing complexities, and integration issues. They also discussed barriers related to corrective actions, objectives, targets, and adapting management systems to change^44^. Vijayaraghavan and Helu (2012) reviewed enabling technologies for green manufacturing, emphasizing the role of sensors in characterizing resource flows and the importance of software frameworks for automated energy and operational data analysis. They further discussed a case study demonstrating energy monitoring for evaluating manufacturing system performance^45^. Onsrud and Simon (2012) highlighted that green manufacturing emerged in response to social, economic, and policy pressures, emphasizing sustainability through metrics, standards, and best practices. They further discussed the key drivers, challenges, and benefits influencing the transition toward sustainable operations^46^. Despeisse et al. (2013) examined sustainable manufacturing by introducing a tactics library and factory modelling approach that linked conceptual sustainability principles with practical resource efficiency practices at the factory level. They emphasized that cross-functional modelling and systematic workflows enabled manufacturers to operationalize sustainability strategies in a structured manner^47^. Govindan et al. (2014) examined the barriers hindering the implementation of green supply chain management in Indian industries and highlighted key challenges through an analytic hierarchy process framework. Their study emphasized the role of expert judgments in identifying critical obstacles that affected the adoption of sustainable practices^48^.

### Adoption of green manufacturing in India

Sangwan et al. (2011) highlighted that the adoption of green manufacturing in Indian SMEs offered both qualitative and quantitative benefits, fostering sustainable industrial practices. They emphasized that managers perceived GM as a viable alternative for improving competitiveness and environmental performance^49^. Minhaj et al. (2012) reviewed the evolution of green manufacturing, highlighting its role across the product life cycle through resource optimization and waste reduction. They emphasized the lack of a unified framework and proposed a comprehensive model to address emerging challenges in sustainability^50^. Minhaj et al. (2013) examined the status of awareness and implementation of green manufacturing practices in medium- and small-scale industries of Vidarbha and developed validated performance measures for assessing environmental manufacturing performance. They concluded that the adoption of GM was at an early stage, requiring greater awareness and industry conviction for its benefits^51^. Digalwar et al. (2013) investigated green manufacturing practices in Indian industries and identified key performance measures through factor analysis. Their study provided an integrative framework that enabled organizations to assess and prioritize green manufacturing initiatives^52^.

### Sustainability drivers in manufacturing

**Fig. 1:**
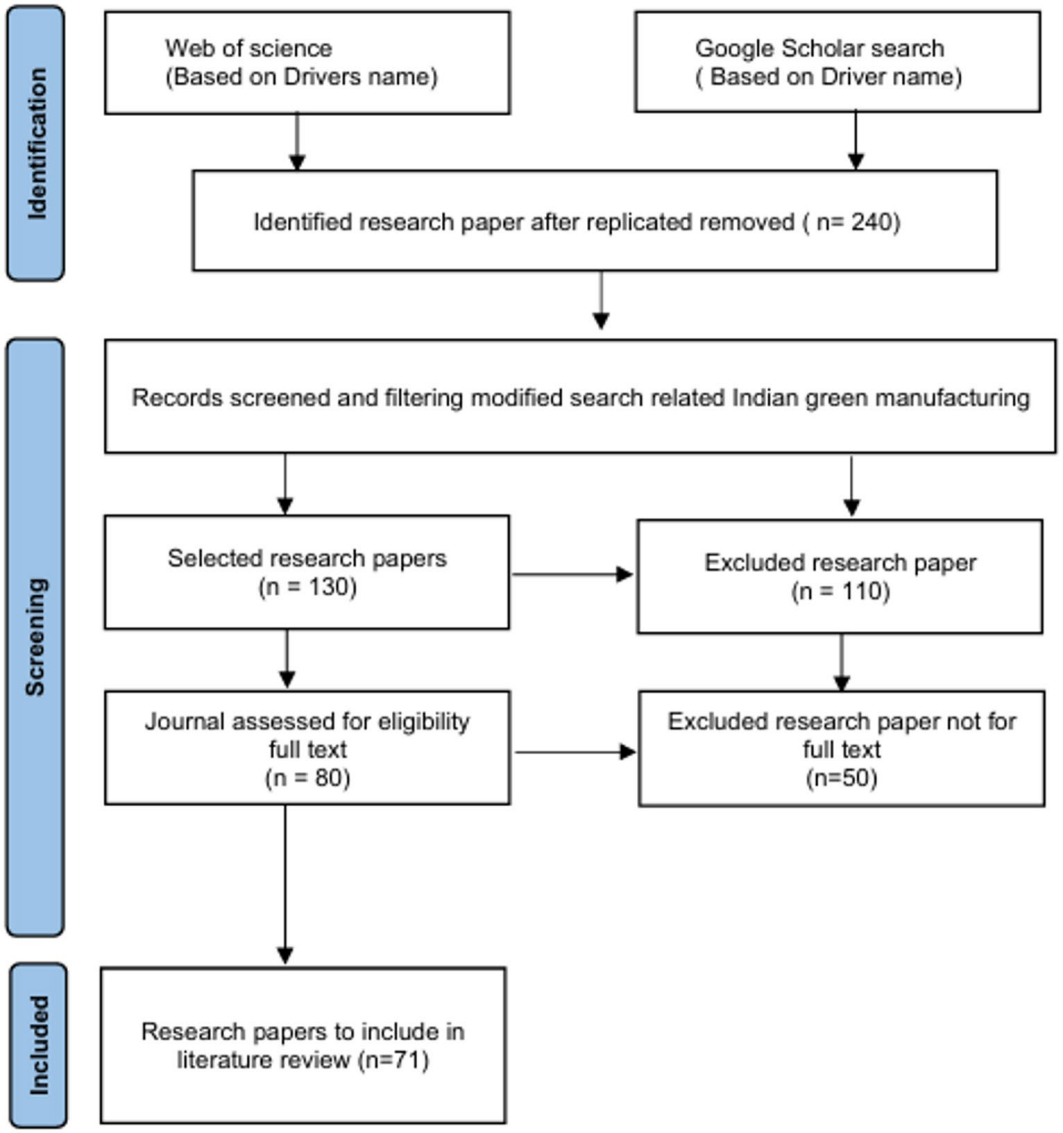
Systematic literature review methodology—PRISMA flow diagram.

Chin et al. (1999) evaluated the critical success factors for implementing ISO 14001-based EMS in Hong Kong manufacturing companies using the Analytic Hierarchy Process and highlighted its role in enhancing environmental performance. They emphasized that despite associated costs, adoption of ISO 14001-based EMS supported long-term competitiveness in the global market^35^. Morrow and Rondinelli (2002) examined the adoption of EMAS certification due to strategic, regulatory, and reputational motivations. They reported that such certifications resulted in improved environmental performance and organizational efficiency across multinational and domestic firms^53^. Ammenberg and Sundin (2005) reported that such integration supported life-cycle perspectives and promoted continuous environmental improvement in manufacturing practices^54^. Gabzdylova et al. (2009) examined sustainability in the New Zealand wine industry and reported that personal values, customer demand, and stakeholder influence shaped the adoption of sustainable practices. They highlighted that wineries engaged in environmental management without necessarily gaining financial premiums from sustainable production^55^. Sarkis et al. (2010) examined the influence of the adoption of environmental practices and reported that this effect was mediated by organizational training. Their study highlighted the complementary role of institutional and resource-based theories in explaining environmentally oriented practices^56^. Massoud et al. (2010) highlighted that the adoption of ISO 14001 in the Lebanese food industry was primarily driven by the need to improve environmental performance and corporate image. They further noted that limited government support, weak stakeholder demand, and the absence of legal requirements hindered effective implementation^57^. Santolaria et al. (2011) examined how eco-design was perceived and integrated within innovation-driven companies in Spain, highlighting its role as a strategic driver of sustainability. They found that eco-design practices were increasingly embedded in organizational strategies and supply chains, shaping future innovation pathways^58^. Zhou et al. (2012) examined the selection and evaluation of green production strategies by proposing analytic and simulation models that supported decision-making under dynamic and uncertain conditions. They emphasized robust model structures to represent process flow, decision logic, and trade-offs in green production applications^59^. Agan et al. (2012) examined the drivers of environmental processes in Turkish SMEs and found that factors such as expected benefits, customer demands, and social responsibility influenced environmental practices. They reported that environmental initiatives like design, disposal, and management systems positively impacted firm performance^60^. Chuang and Yang (2013) highlighted that the implementation of a green manufacturing system was strongly influenced by green design, eco-compliance, and pollution reduction strategies. Their study demonstrated that success factors were shaped by material choices, regulatory alignment, and environmentally conscious processes^61^. Sharma et al. (2024) examined circular economy practices in Indian manufacturing and identified multiple enablers and barriers influencing adoption^65^. The systematic process employed in this investigation for the identification of the relevant publications present is shown in Fig. 1. The data reported in Fig. 1 are obtained using the standard procedure called PRISMA (Preferred Reporting Items for Systematic Reviews and Meta-Analyses), which is a standard process that guides systematic review reporting.

The expert panel involved of 18 professionals, including senior production managers, environmental compliance officers, and quality control Uttar Pradesh engineers representing textile, automotive, chemical, food processing, and electronics manufacturing sectors. Their industrial experience ranged from 5 to 22 years, and they were drawn from major industrial groups across Uttar Pradesh (Kanpur, Lucknow, Ghaziabad, Varanasi, and Gorakhpur).

The current research addresses these gaps by finding common and essential drivers in green manufacturing and developing a systematic framework for their evaluation. By doing so, it aims to provide actionable insights for Indian manufacturers to integrate green manufacturing into their operational strategies, ensuring both environmental sustainability and competitive advantage. This research is particularly relevant as industries worldwide move toward greener practices, and India emerges as a significant contributor to global manufacturing sustainability.

## Research gap and objectives

It is concluded from above review that there is no comprehensive work addressing practical implementation of green manufacturing through validated models.

Very few researchers have investigated concept, but their focus remains largely theoretical, with limited assessment frameworks. Studies exploring driver identification often restrict their analysis to basic aspects such as financial gains and regulatory compliance, without prioritization or in-depth evaluation. Moreover, very few works are regional, particularly in context of Indian manufacturing industries. Existing research also lacks validation of proposed models in real industrial environments. Hence, this study attempts to fill this gap by identifying and prioritizing drivers of green manufacturing and validating the findings in the Indian manufacturing context.

The research questions are as follows:

- What drives green manufacturing adoption in Indian manufacturing?
- What kind of results can Random Forest Regression quantify and rank drivers?
- Which drivers are most important for green manufacturing?
- How does the dual-phase framework connect theory and practice?

The main objective of this research is to develop a comprehensive and systematic model for implementing green manufacturing. The proposed framework integrates transformation phases, planning targets, controllers, and strategies, while identifying and prioritizing key drivers through surveys and data analysis. Using regression forest models and study validates framework for practical applicability, addressing existing literature gaps with a structured approach for assessment and implementation.

The following objective point of view below:

- To develop an AI-enabled framework for implementing green manufacturing by integrating performance evaluation with driver identification.
- To identify and prioritize critical drivers of green manufacturing through regression forest modelling and industrial feedback.
- To validate proposals through Indian manufacturing organizations to ensure practical relevance and applicability.

The novelty of present paper lies in developing a model for green manufacturing and a model integrating driver identification through a regression forest model. It highlights key drivers, validates model in Indian industrial contexts, and provides an organized route through the gap between theory and implementation.

## Problem statement

The manufacturing sector in India is under growing pressure to transition towards sustainable practices due to escalating environmental concerns, resource scarcity, and global regulatory trends. Green manufacturing has emerged as a strategic response, aiming to optimize resource and energy use and minimize waste. It also seeks to reduce ecological impact while maintaining competitiveness. Green manufacturing has the potential to improve operational efficiency, enhance brand reputation, and meet stakeholder expectations. However, its practical adoption in India remains inconsistent. Most existing studies offer either theoretical discussions or fragmented, region-specific findings. They lack a unified, data-driven approach to identify and prioritize the most influential green manufacturing drivers in the Indian industrial context. Current green manufacturing practices are generally implemented through green energy, green products, and green processes.

Indian manufacturing companies lack a defined framework for identifying and prioritizing high-impact green manufacturing drivers despite rising sustainability demands. Existing studies lack local industrial validation and are conceptual and dispersed. Lack of an organized, data-driven prioritization tool hinders managerial resource allocation and policy alignment. Thus, this effort aims to produce and evaluate a driver identification framework that incorporates expert judgment, industrial surveys, and Random Forest modeling to help green manufacturing decision-making. The process ranked and validated Indian green manufacturing drivers.

### Proposed research framework

**Fig. 2:**
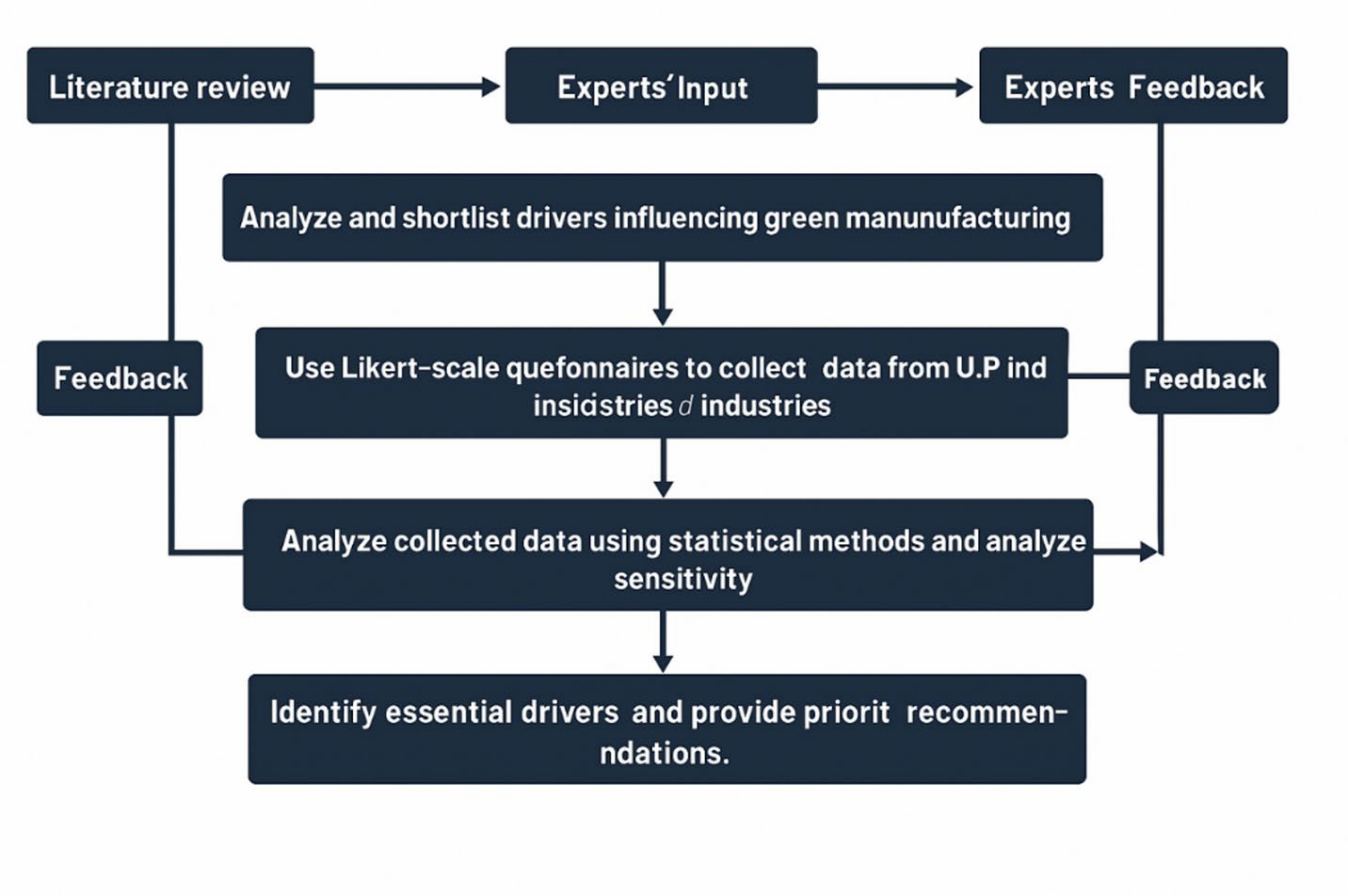
Approach of the identification of essential drivers.

Through an extensive literature search, expert discussions, and scheduled industrial feedback sessions, the key determinants of green manufacturing were identified. The literature review used simply to establish an initial driver pool, which was further modified and independently validated by expert judgment and consultations with manufacturing specialists in Uttar Pradesh to ensure contextual relevance in India. Utilizing Random Forest regression on data sourced from industries across various cities in Uttar Pradesh, India, the essential drivers and their relative importance have been concluded. Figure 2 illustrates proposed study framework. The suggested investigation study is validated through a dual-phase approach. The initial phase entails the development of questionnaire that identifies primary drivers of green manufacturing. The subsequent phase involves implementation of Random Forest regression method with MATLAB software to ascertain priority and essentiality of each driver.

## Methodology

In general, data-driven decision-support frameworks have proven effective in solving complex industrial problems by integrating structured modelling with analytical evaluation techniques^1,2^. Among such tools, ensemble-based machine learning methods particularly Random Forest regression have gained attention for their robustness, predictive accuracy, and capacity to identify variable importance^18,59^. Originally introduced as an enhancement over conventional decision trees, Random Forest applies bootstrap aggregation and random subspace selection to construct multiple learners, combining their outputs to reduce overfitting and improve stability^18,59^. This capability makes it particularly suitable for industrial decision contexts where variables are interdependent, and the dataset may contain uncertainty^64^. While traditional ranking methods, such as linear statistical models or purely judgment-based approaches, have been widely used to evaluate drivers of green manufacturing, they often assume linearity and can be biased by subjective assessments^49,51^. In contrast, Random Forest inherently accommodates non-linear interactions among predictors and integrates feature importance estimation, offering a more objective and data-driven basis for prioritization^64^. This is essential in sustainability-focused manufacturing systems where influencing factors ranging from economic benefits and regulatory compliance to technological readiness and stakeholder expectations are rarely independent^52,54^. The proposed methodology combines theoretical insights with practical industry validation.

### Implementation of proposed framework

The proposed framework is validated through a two-phase approach. The first phase focuses on identifying the twelve key drivers of green manufacturing through literature review, expert consultations, and industrial feedback across multiple sectors in Uttar Pradesh, India. This stage establishes a comprehensive driver set covering economic, environmental, legislative, and stakeholder aspects. The second phase applies Random Forest regression model in MATLAB to prioritize and quantify the relative importance of these drivers. Industrial survey data is processed, and model outputs are validated using statistical metrics (R^2^, MAE, RMSE) and visual tools such as scatter plots, heatmaps, and importance charts. Expert reviews from participating firms further confirm the operational relevance of the ranked drivers. This dual-phase application ensures that the framework is both data-driven and industry-validated, enabling manufacturing organizations to strategically focus on high-impact drivers for effective green manufacturing implementation.

### Identification of green manufacturing drivers

First, through a review of existing literature and insights from industry and field specialists in Uttar Pradesh, India. This paper identifies the key drivers of green manufacturing using two distinct approaches. These specialists hailed from cities including Banaras, Agra, Kanpur, Lucknow, Gorakhpur, and Ghaziabad. The study began with a comprehensive review of current research in areas such as green manufacturing, sustainable practices, corporate image value, and financial advantages. Subsequently, key elements of green manufacturing were gathered and analyzed in several stages of discussion, based on findings from the literature review, expert opinions, and industrial feedback. The research terms, including "sustainable manufacturing," "sustainable practices," "conscious manufacturing," "factors of green manufacturing," and "green manufacturing facilities in the Indian context," were used. These were sourced from reputable publications such as Wiley, Springer, Elsevier, and Taylor & Francis, among others, to identify relevant literature on the topic. Additionally, the program focused on “green manufacturing” was conducted to engage 100 industrial production executives from various industrial hubs in Uttar Pradesh, including Kanpur, Banaras, Ghaziabad, and Gorakhpur, for four months. Among these, 95 executives participated in offline sessions. The program reinforced the importance of green manufacturing in the Indian context. After explaining the subject, participants engaged in multiple rounds of discussions and clarifications. This dual approach of literature review and professional expertise helped bridge the research gap between theoretical knowledge and practical insights. Ultimately, based on the literature review, expert opinions, and feedback from industrial executives, twelve fundamental drivers of green manufacturing were finalized for identification.

Thus, the identification of drivers followed a multi-stage and independent process involving literature synthesis, expert consultation, and direct industrial engagement. This approach ensured that the finalized twelve drivers were not merely adopted from prior studies but emerged through contextual validation and practical relevance to Indian manufacturing conditions, thereby minimizing respondent bias and predefined prompting.

The most prevalent drivers were determined after numerous sessions of communicates and clarifications of green manufacturing, as illustrated in Table 1.

**Table 1:** Most prevalent drivers for green manufacturing.

Symbol	Drivers name	Details	Revised references
D1	Economic benefits	The economic benefits to adopt green manufacturing since these methods ensure optimum use of resources and energy, thereby increasing the financial gains for the company	^55^
D2	Business reputation	Integrity is essential for organizational growth; therefore, green manufacturing practices help maintain and enhance business reputation	^52^^,^^48^
D3	Environmental issues	Implementation of green manufacturing is motivated by depletion of natural resources and rising environmental concerns	^64^^,^^34^
D4	Agreement and legislation	To comply with laws such as ISO 14001 and other legislative requirements, companies are compelled to maintain sustainable practices	^62^^,^^44^
D5	Stakeholders	Investors, media, and government influence organizational decisions. Stakeholder pressure has recently driven the adoption of green manufacturing	^55^^,^^52^
D6	Sustainable novelty	Increasing focus on sustainable development has encouraged manufacturers to adopt innovative green strategies	^48^^,^^46^
D7	Logistical needs	Reverse logistics and supply chain expansion frequently drive manufacturers to design eco-friendly, recyclable, and easily disassembled products	^37^^,^^63^
D8	Prospective client	With increasing environmental awareness, customers are demanding more sustainable products	^54^
D9	Employee requests	Workers expect companies to adopt green systems because certain operations may harm both employees and the environment	^44^
D10	Internal drivers	Adoption of green manufacturing enhances employee loyalty by creating a positive environmental culture	^62^
D11	Economic conditions	Economic pressures and external support encourage manufacturers to produce green products through sustainable operations	^55^^,^^63^
D12	Competitors in industries	To remain competitive, firms must introduce new green concepts, which often provide strategic advantages	^62^^,^^52^^,^^44^

### Random Forest regression model

The evaluating procedure for this methodology is described as follows:

***Step 1:*** Identify the common attributes (drivers) related to green manufacturing with the combined assistance of existing literature, field and industrial experts’ opinions.

***Step 2:*** Prepare data matrix and target vector from questionnaire responses and performance scoring. Convert Likert responses into numeric feature values and assemble the dataset as:

$$X = \left[ {\begin{array}{*{20}c} {x_{11} } & { x_{12} \ldots \ldots \ldots } & {x_{1p} } \\ {x_{{\begin{array}{*{20}c} {21} \\ . \\ . \\ . \\ . \\ . \\ . \\ . \\ \end{array} }} } & { x_{{\begin{array}{*{20}c} {22} \\ . \\ . \\ . \\ . \\ . \\ . \\ . \\ \end{array} }} \ldots \ldots \ldots } & {x_{{\begin{array}{*{20}c} {2p} \\ . \\ . \\ . \\ . \\ . \\ . \\ . \\ \end{array} }} } \\ {x_{n1} } & {x_{n2} } & {x_{np} } \\ \end{array} } \right],\;\;\;y = \left[ {\begin{array}{*{20}c} {y_{1} } \\ {y_{2} } \\ . \\ . \\ {y_{n} } \\ \end{array} } \right]$$

where *n* is number of firms (observations) and *p* = 12 is number of drivers (features). The dependent variable (*y*) is green manufacturing success score (obtained through expert evaluation).

***Step 3:*** Data preprocessing normalizes features, check and remove incomplete responses, split into training and test sets (80:20). Optionally use k-fold CV for robust performance estimates.

***Step 4:*** Train random forest regression and obtain ensemble predictor. For *K* trees random forest predictor is^64^:

1 $${\text{Random forest regression }} = { }\frac{1}{K}\mathop \sum \limits_{k = 1}^{K} h_{k} \left( x \right)$$

where ℎ_*k*_ is prediction of *k*-th regression tree. Key hyperparameters used: number of trees.

*K* maximum depth, minimum samples per leaf, and bootstrap sampling.

In this study, the Random Forest regression approach was adopted to quantify the relative significance of twelve key drivers of green manufacturing identified through an extensive literature review and expert consultations across industrial hubs in Uttar Pradesh, India (Kanpur, Banaras, Ghaziabad, Gorakhpur). Each driver was evaluated on a nine-point Likert scale, reflecting its perceived influence on green manufacturing outcomes. All input data were normalized via min–max scaling to ensure comparability.

The Random Forest model was implemented in MATLAB with parameters set to 100 trees, a maximum depth of five, and bootstrap sampling enabled. The dataset was partitioned into training (80%) and testing (20%) subsets to assess generalization performance. Out-of-bag (OOB) error estimation was used to calculate predictor importance, which was then normalized to determine the relative weight of each driver. Evaluation metrics included the coefficient of determination (R^2^), mean absolute error (MAE), root mean squared error (RMSE), maximum absolute error, and median absolute error^64^.

Random Forest Regression was selected due to its robustness against overfitting, ability to model non-linear interactions between input drivers, and built-in feature importance extraction, which is crucial for prioritizing green manufacturing drivers. It is particularly effective for low-dimensional structured data, as in this study (n = 45, p = 12) as listed in Table. 2.

**Table 2:** Random Forest regression model parameters used in current study.

Parameter	Value
Number of trees	100
Max depth	5
Min samples/leaf	2
Bootstrap sampling	Yes
Train-test split	80:20

***Step 5:*** Feature (driver) importance extraction. Use OOB (out-of-bag) or permutation importance. Permutation importance for feature *j* can be computed as:

***Step 6:*** Model validation and stability check a cyclic process until acceptable performance is reached. The steps in the cycle are:

Reducing variance in ensemble random forest regression model and avoiding overfit depend on bagging. The learner trees so must not be connected. Collated from original training dataset, the samples not chosen for training kth regression tree during bagging create an out of bag data dataset. OOB often corresponds to one-third of *D*. The mean squared error (*MSEO*O*B*) of the *k*th regression tree is obtained from OOB dataset as^64^^,^

2 $${\text{Mean squared error }} = { }\frac{1}{n}\mathop \sum \limits_{i = 1}^{n} (y_{i} - \overline{y}_{iOOB} )^{2}$$

where the *i*th prediction and mean of *i*th forecast from all trees are denoted by $y_{i}$ and $\overline{y}_{iOOB}$.

The following relation can be used to calculate the coefficient of determination, or *R*^2^*OOB*, of the OOB dataset from *MSE*_*OOB*_ based on the total variance, or *Var*_*y*_.

3 $$R_{OOB}^{2} = 1 - \frac{{MSE_{OOB} }}{{Var_{y} }}$$

The random forest regression model predicts an appropriate outcome response. by $\hat{y}_{i}$, whereas driver response is represented by $y_{i}$. The residual $\varepsilon_{i}$ can therefore be calculated as^64^:

4 $$\varepsilon_{i} = y_{i} - \hat{y}_{i}$$

The R^2^ (coefficient of determination) measures how well a regression model explains variability of target variable.^64^:

5 $$R^{2} = 1 - \frac{{\mathop \sum \nolimits_{i = 1}^{n} (y_{i} - \hat{y}_{i} )^{2} }}{{\mathop \sum \nolimits_{i = 1}^{n} (y_{i} - \overline{y})^{2} }}$$

The mean absolute error can be calculated by considering the total number of samples (n). Likewise, the mean squared error, maximum error, median error, and root mean squared error are calculated as^64^:

6 $$Mean absolute error = \frac{{\mathop \sum \nolimits_{i = 1}^{n} \left| {y_{i} - \hat{y}_{i} } \right|}}{n}$$

7 $$Mean squared error = \frac{{\mathop \sum \nolimits_{i = 1}^{n} (y_{i} - \hat{y}_{i} )^{2} }}{n}$$

8 $$Root mean squared error = \sqrt {\frac{{\mathop \sum \nolimits_{i = 1}^{n} (y_{i} - \hat{y}_{i} )^{2} }}{n}}$$

***Step 7:*** Industrial validation present ranked drivers and model diagnostics to participating industrial experts for plausibility checks and feedback. Incorporate expert feedback (if necessary) and report final prioritized driver list.

### Data collection and questionnaire preparation

The dataset used in this study was collected from 100 Indian manufacturing firms representing various sectors, including iron, textiles, electronics, food, and chemicals. Each data point corresponds to a single firm and includes 12 independent input variables (drivers D1 to D12). The dependent variable, or output label, is the Green Manufacturing Success Score, which was obtained using performance evaluations, expert judgment, and industry benchmarking. Out of 100 participating firms, 45 complete responses were retained for modelling due to strict completeness and consistency criteria. Given the exploratory objective of driver prioritization rather than statistical generalization, such sample sizes are acceptable in sustainability-driven industrial studies. Nonetheless, results must be interpreted cautiously when extrapolating beyond the surveyed regions. This score ranges from 50 to 100 and serves as prediction target for Random Forest Regression model (Annexure-1, supplementary file).

All input features were treated as ordinal variables and normalized using min–max scaling for model compatibility. No missing data imputation was necessary as only fully completed responses (n = 45 used for modeling) were retained. A questionnaire was developed using the twelve key drivers determined through the advice of experts, industrial executers, and preceding publications.

A questionnaire based on the drivers listed in the paper:

1. Rate the importance of Economic benefits (D_1_) in adopting green manufacturing.
2. Rate the importance of Business reputation (D_2_) in adopting green manufacturing.
3. Rate the importance of Environmental issues (D_3_).
4. Rate the importance of Agreement and legislation (D_4_).
5. Rate the impact of stakeholders (D_5_).
6. Rate the importance of Sustainable novelty (D_6_).
7. Rate the importance of Logistical needs (D_7_).
8. Rate the impact of Prospective client (D_8_).
9. Rate the impact of employee requests (D_9_).
10. Rate the impact of Internal drivers (D_10_).
11. Rate the impact of Economic conditions (D_11_).
12. Rate the impact of Competitors in industries (D_12_).

The questionnaire design was finalized after expert review and pilot industrial discussions to ensure clarity and neutrality of questions. The drivers included were contextually validated and framed to independent industrial perception rather than enforcing predefined theoretical assumptions.

### Random regression analysis

**Fig. 3:**
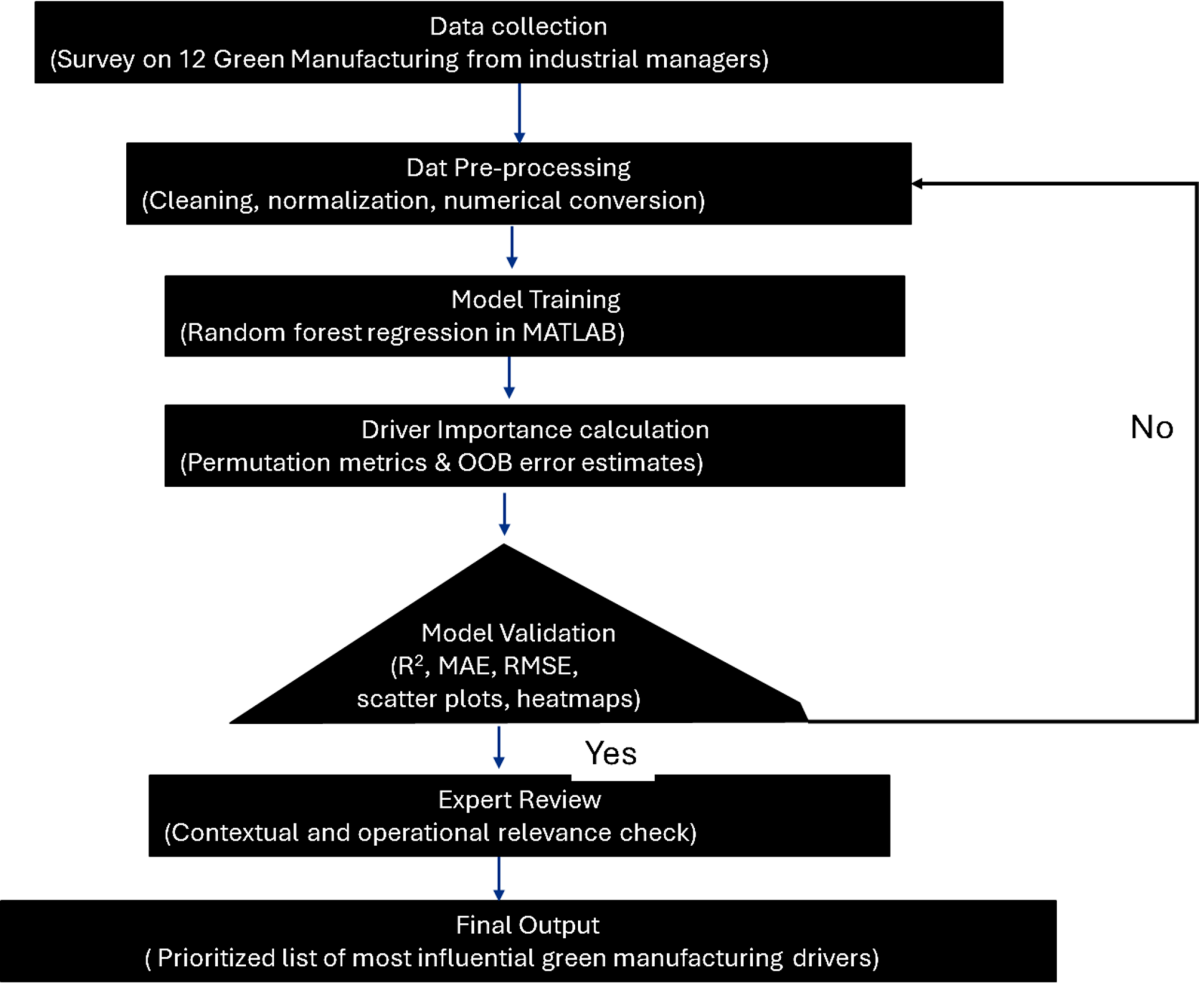
Steps involved in Random Forest regression approach.

MATLAB random regression investigates industry data, which is represented in Fig. 3. The four-step technique from previous sections, priority among main drivers is identified. The following steps are below.

1. Main drivers were collected and identified of green manufacturing in Uttar Pradesh, India.
2. To simulate drivers scores and success target for green manufacturing
3. 12 drivers selected and target income score (50- 100)
4. Splits the data into training and testing with 80%and 20% subsets for model validation.
5. Trains a 100-tree random forest model with regression method on MATLAB.
6. Predictive ability to estimate target scores from main drivers
7. Calculate mean absolute error, mean squared error, root mean squared error, maximum error, and median error for predictions versus actual found.
8. Normalized weights are computed using OOB predictor importance of drivers from the random forest regression method.
9. Include the graphical explanations through driver contribution levels, scatter plots, cox plot of driver score distribution, heat map for visualizations and histogram of target values.

## Develop a model enabling implementation of green manufacturing

To effectively implements green manufacturing, it is important to develop systematic and structured model. This model should aim to create a high-level framework that integrates various subsystems and related components within the manufacturing process. By establishing these interconnections, the framework enables a comprehensive understanding of how different elements contribute to green manufacturing goals, supporting more informed decision-making and efficient strategy development for sustainable industrial practices. Thus, before implementing the proposed model, following aims must be clearly understood:

- To assess the requirements of green manufacturing across various fields and activities.
- To transform green manufacturing requirements into measurable tools for control and implementation.
- To ensure sustainable development by building an economical and enviro- friendly system.

**Fig. 4:**
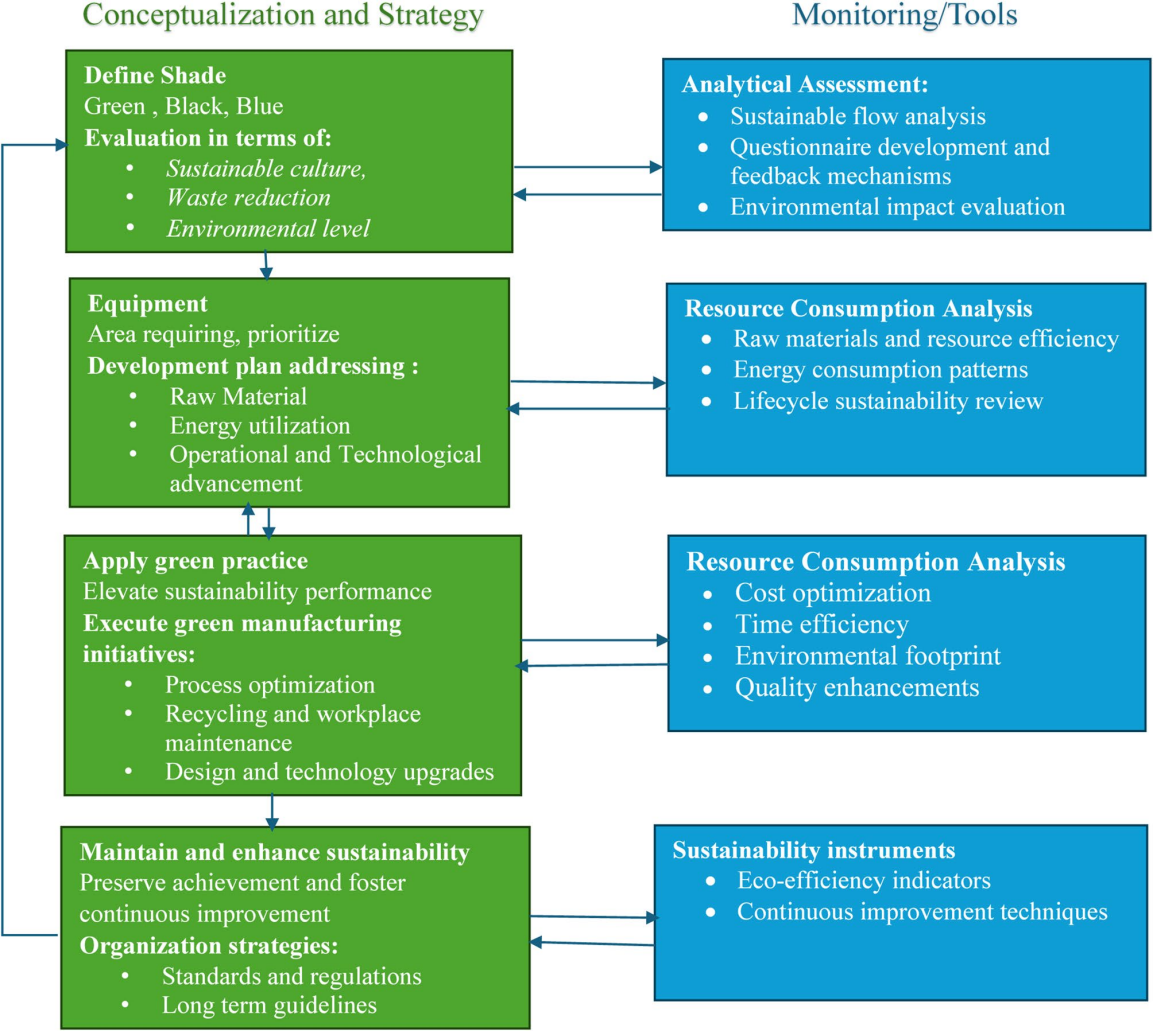
Develop model of green manufacturing^48^.

Figure 4 illustrates the green manufacturing model for design and construction under controlled conditions. The model consists of two modules: the first focuses on conceptualization and Strategy, while the second emphasizes monitoring. The control module of model is based on performance assessments, reflecting main objectives and constraints necessary for decision-making at each level^45^.

The developed model is open to information, with data flow accessible through any layer or component, as it integrates both order and separate levels. The model’s development is divided into four parts, that is represents in the following sections.

### Performance evaluation

The main objective is to promote sustainability and minimal waste using resources in field of green manufacturing. Any improvement process begins with an evaluation of current scenario, aiming to identify pathways for transitioning toward green manufacturing system. This layer of green manufacturing conceptualization and strategic planning involves assessing greenness across various levels, ranging from operational processes to system-wide structures. The primary challenge lies in developing a robust quantitative evaluation framework, as meaningful improvements are not possible without precise measurements^49^.

**Fig. 5:**
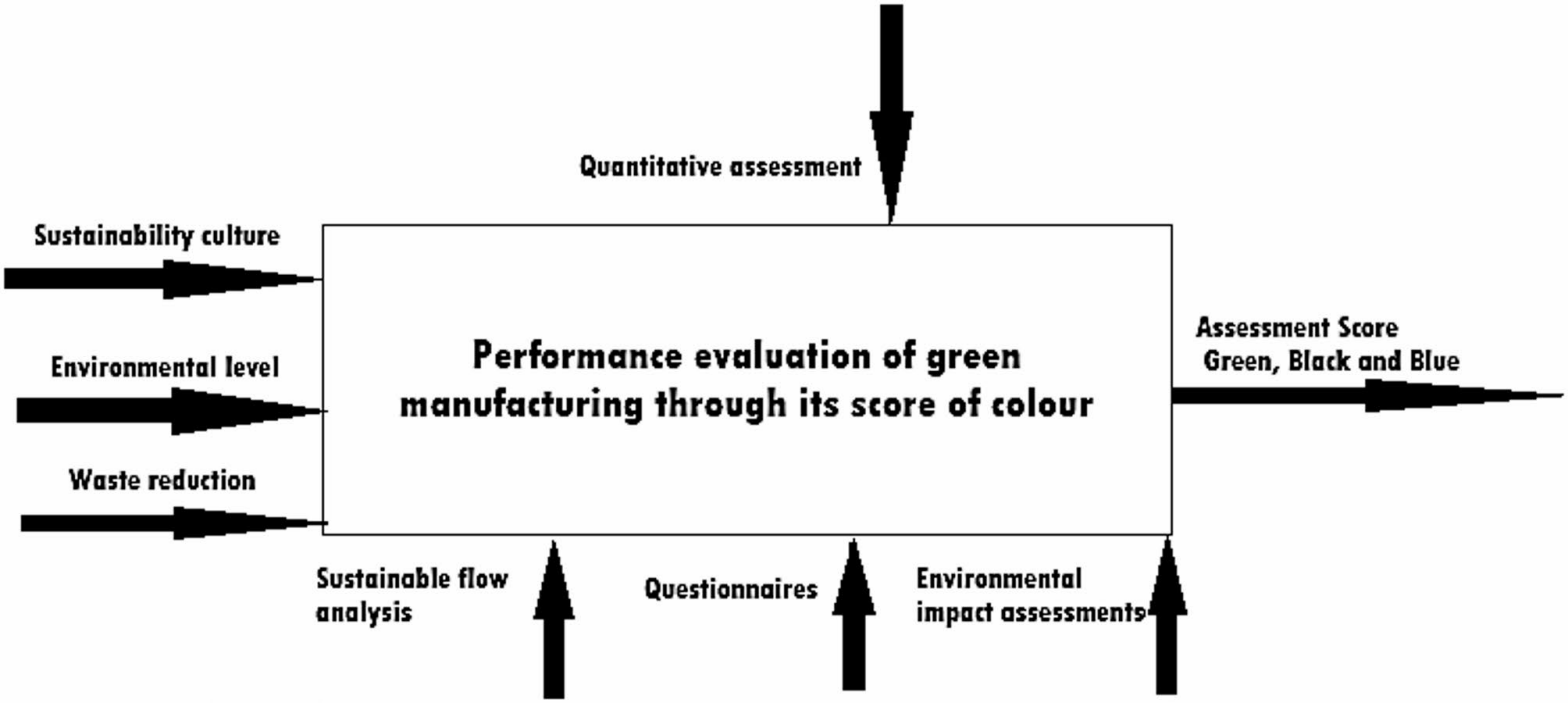
A systematic flow of Performance evaluation layer^50^.

In Fig. 5 represents that the evaluation process must adopt a multidimensional approach, leveraging advanced tools and methodologies to ensure an accurate and comprehensive quantification of greenness. In green manufacturing assessments, symbolic colours such as green, black, and blue are utilized to represent varying degrees of sustainability. The green colour denotes complete sustainability, black signifies that the system is far from being sustainable, and blue indicates intermediate stages, emphasizing partial greenness and areas requiring improvement^56^. These colours are linked to a corresponding quantitative value, which is used to determine the system’s greenness score. According to model, the green manufacturing metric is quantitative evaluation measure that assesses three key dimensions of green manufacturing : sustainability culture, environment level, and waste reduction:

- **Sustainability culture:** It involves qualitative measurement of workers’ green practices and awareness activities.
- **Environmental level:** This evaluates environmental impact, pollution, and carbon footprint.
- **Waste reduction:** This measures how much raw material and energy are wasted and how much is recycled within the system.

Methods such as planned interviews, questionnaires, environmental impact assessments, and Sustainable Flow Analysis (SFA) are employed to evaluate these factors. SFA, based on flow analysis, recognizes sustainability prospects within the manufacturing system. It provides benchmarks for evaluating performance and establishing enhancement objectives throughout sustainability transition process^56^. Neural networks, artificial intelligence, and fuzzy logic systems will be incorporated into the development of SFA. These approaches assist the measurement and analysis of both quantitative and qualitative information. This metric aims to assess current conditions and help with the improvement process. The fundamental principle of evaluation process is that green manufacturing can only enhance through kind of measurements. A systematic and quantitative strategy is employed to evaluate greenness. The green index derived from metric guides green transition process, promoting connection with green benchmark objectives^54^.

This green manufacturing model promotes industries to pursue environmental sustainability by actively engaging in waste management, pollution control, and the implementation of eco-friendly practices. Through techniques such as Sustainable Flow Analysis, industries can enhance their performance while simultaneously laying a robust foundation for enduring sustainability. This process reduces environmental impacts and promotes industries to adopt sustainable methods, thus providing a balanced and sustainable future.

### Development planning

In this layer, a plan is generated before implementing green manufacturing, based on the foundation of the previous performance evaluation layer. The planning phase plays a significant role in maintaining production levels in alignment with market demand. The importance of this layer also lies in ensuring that the improvements and implementations in green manufacturing do not negatively impact the productivity assumed during performance evaluation^55^. The primary aim of planning should be to prioritize areas accordingly. Planning and preparation should be continued out at machine level, where prioritization decisions are made based on assessment scores at process level and system level, considering present order. During development, focus should be divided into three parts in terms of quality and quantity:

- Raw Material
- Energy Utilization – determining where and how it is being used
- Operational and Technical Advancements

**Fig. 6:**
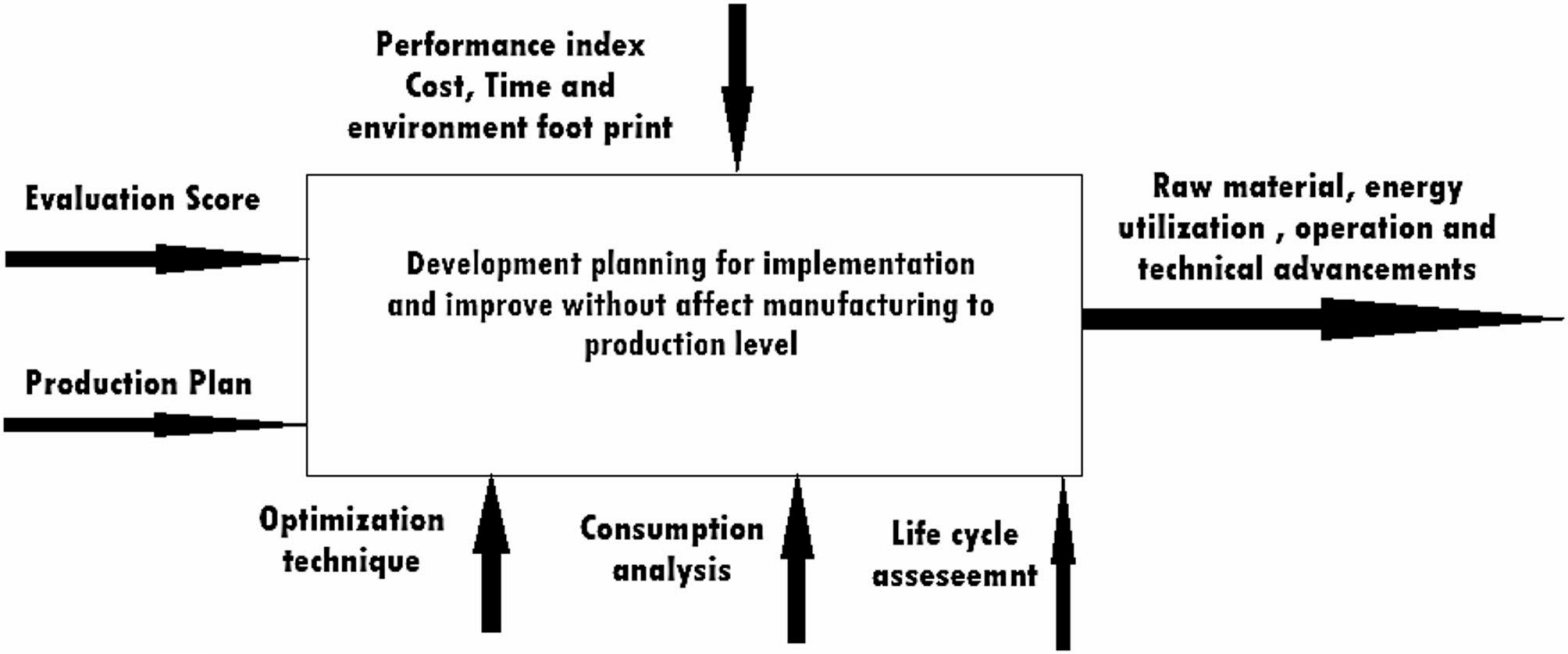
A systematic flow of planning of green manufacturing layer^56^.

Due to the varied nature of objectives, the planning of green manufacturing at this stage primarily revolves around the optimization process as shown in Fig. 6. The planning of green manufacturing aims to maintain low costs while minimizing raw material and consumption of energy during development process. This serves as the fundamental objective for a comparative assessment analysis related to material, manufacturing costs, and productivity at minimal expenses. An optimal plan must be determined to align market demand with greener productivity, without compromising on quality, quantity, or the given timelines. The plan will be executed by translating optimal raw material usage and minimal energy reduction levels into modified requirements, optimal process parameters, and control parameters through the insights gained from the previous layer of performance evaluation.

### Execute green manufacturing (Implementation)

**Fig. 7:**
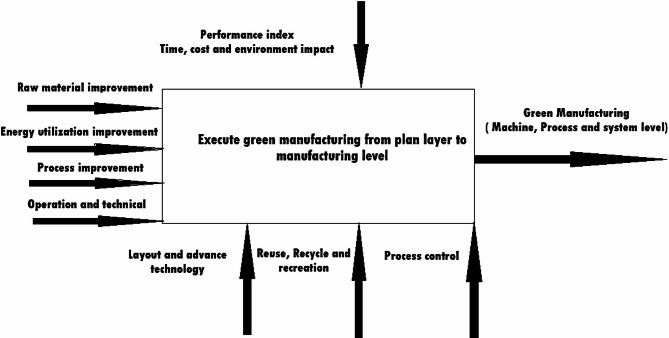
A systematic flow of green manufacturing implementation layer^59^.

After development of an optimal plan, the next step is gradual execution and implementation of green manufacturing as represents in Fig. 7^58^. The development of planning through raw material, energy reduction, and operational with technology is decomposed and executes green manufacturing either concurrently or separately with all other aspects. To execute green manufacturing, a framework methodology needs to be established, considering the current situation’s demand practically and on a market basis. Then, after planning development based on the performance evaluation conducted in first layer, gradually transmitted formation of this implementation or execution is done. Among all these, it must be determined that productivity will not be affected from the first to the implementation phase^59^.

At each layer, to execute the green manufacturing plan, a re-evaluation process should be done using the developed green manufacturing metric. The re-evaluation process will measure how much improvement has been achieved at the manufacturing level, starting from the plan layer of the first phase, and judge at each level whether greener improvement has been successfully achieved or not.

### Maintenance enhances and sustainability

This fourth Layer determines, how much improvement has been achieved in the green manufacturing, with objective of enhancing maintenance and elevating level of sustainability. The goal of green manufacturing is to ensure that sustainability integrated into each layer and component. From initial raw materials to the manufacturing level, enhancing maintenance is the primary objective at this stage^60^.

**Fig. 8:**
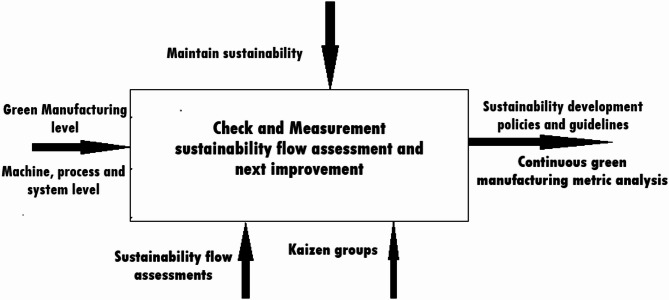
A systematic flow of sustainability and maintenance enhance layer^61^.

In Fig. 8 illustrate that the expected outcomes include the development of long-term policies, guidelines, and standards. Enhancing maintenance implies the continuous measurement of sustainability levels across various stages, such as selection, evaluation, and implementation. Green Kaizen groups should be incorporated as part of planning and control activities to improve the green manufacturing system^45,61^. The biggest challenge at this present stage of green transformation is typically dynamic controlling throughout process. Continuous measurement of sustainability (or greenness) should act as a trigger for other green improvement plans, ensuring that the process continues to evolve without interruption^52^.

### Monitoring

Green manufacturing relies on monitoring tools and analytical assessment methods to ensure sustainable development throughout manufacturing to production level. Sustainable flow analysis tracks movement of raw materials, energy reduction, highlighting opportunities for improvement. The framework of questionnaire and feedback gather stakeholder insights on challenges and enhancements^54^. The performance assessments environmental impact, resource consumption analysis measure emissions, water uses, and waste reduction while identifying resource. The focus on raw materials and resource ensures low waste and promotes renewable resources^55^. The energy consumption is analysed to identify high energy use areas, supported by sustainability like energy audits and carbon accounting. To foster continuous progress, Kaizen and Lean manufacturing techniques are applied for incremental process levels. Lifecycle sustainability assesses product process from raw material extraction to productivity, identifying sustainability gaps^56^.

Simultaneously, cost optimization and time efficiency strategies reduce operational costs and production time while maintaining quality and sustainability. The Tools calculate carbon footprint, water usage, and ecological impact, driving mitigation strategies.

By monitoring and tools methods, green manufacturing achieves measurable improvements in environmental, resource usages, and operational maintenance. Continuous monitoring ensures alignment with sustainability, while feedback and data driven insights drive innovation and ongoing enhancements across all stages of the manufacturing.

## Results and discussion

Table 3 presents normalized feature importance values for all twelve identified drivers of green manufacturing as determined through Random Forest regression model. The values represent the contribution of each driver toward predicting green manufacturing success, calculated using the mean decrease in impurity (MDI) approach. Higher values in a row signify that the driver in that row has greater predictive influence when considered in conjunction with the column driver. For example, D4 (Agreement and Legislation) shows consistently high comparative importance values (0.145) when evaluated against most other drivers, confirming its dominant role in shaping green manufacturing outcomes. Patterns in the table indicate that D1 (Economic Benefits), D5 (Stakeholders), and D8 (Sustainable Novelty) also achieve relatively high importance scores across comparisons, suggesting that financial gains, stakeholder involvement, and innovation remain central to successful green manufacturing adoption. In contrast, D9 (Employee Requests) and D10 (Logistical Needs) display lower values (0.056 and 0.049), which reflects their comparatively limited predictive power in the model. These relative importance values form the basis for deriving the final ranked list of drivers shown in Table 3. The results confirm that regulatory alignment, economic incentives, and stakeholder-driven initiatives are key levers for advancing green manufacturing performance, while internal operational factors such as employee requests or logistical constraints, though relevant, play a secondary role in influencing success.

**Table 3:** Pair wise comparison of drivers.

	D1	D2	D3	D4	D5	D6	D7	D8	D9	D10	D11	D12
D1	(1,1,1)	(1/4,1/3,1/2)	(1/3,1/2,1)	(3,4,5)	(4,5,6)	(2,3,4)	(2,3,4)	(1/3,1/2,1)	(4,5,6)	(4,5,6)	(1/3,1/2,1)	(1/3,1/2,1)
D2	(2,3,4)	(1,1,1)	(1/3,1/2,1)	(3,4,5)	(3,4,5)	(2,3,4)	(2,3,4)	(1/3,1/2,1)	(6,7,8)	(3,4,5)	(1/3,1/2,1)	(1/3,1/2,1)
D3	(1,1.5,3)	(1,2,3)	(1,1,1)	(1,3,4)	(4,5,6)	(1,2,2)	(1,2,3)	(1,3,4)	(3,4,5)	(2,3,4)	(1,3,4)	(1,3,4)
D4	(1/3,1/4,1/5)	(1/3,1/4,1/5)	(1,1,1)	(1,1,1)	(4,5,6)	(3,4,5)	(2,3,4)	(1,2,3)	(8,9,10)	(2,3,4)	(1,2,3)	(1,2,3)
D5	(1/4,1/5,1/6)	(1/3,1/4,1/5)	(1/4,1/5,1/6)	(1/4,1/5,1/6)	(1,1,1)	(2,3,4)	(2,3,4)	(1,2,3)	(1,3,4)	(1,2,3)	(1,2,3)	(1,2,3)
D6	(1/2,1/3,1/4)	(1/2,1/3,1/4)	(1/2,1/3,1/4)	(1/3,1/4,1/5)	(1/2,1/3,1/4)	(1,1,1)	(1,2,3)	(1,2,3)	(2,3,4)	(2,3,4)	(1,2,3)	(1,2,3)
D7	(1/2,1/3,1/4)	(1/2,1/3,1/4)	(1/3,1/4,1/5)	(1/4,1/5,1/6)	(1/2,1/3,1/4)	(1/2,1/3,1/4)	(1,1,1)	(2,3,4)	(2,3,4)	(2,3,4)	(1/3,1/4,1/5)	(1/3,1/4,1/5)
D8	(1,3,4)	(1,2,3)	(1,3,4)	(1/2,1/3,1/4)	(1/3,1/4,1/5)	(1/3,1/4,1/5)	(1/4,1/5,1/6)	(1,1,1)	(1,3,4)	(1,1,2)	(1,2,3)	(1,3,4)
D9	(1/4,1/5,1/6)	(1/6,1/7,1/8)	(1/3,1/4,1/5)	(1/8,1/9,1/10)	(1/4,1/5,1/6)	(1/2,1/3,1/4)	(1/2,1/3,1/4)	(1/3,1/4,1/5)	(1,1,1)	(1,1,2)	(1,2,3)	(1,2,3)
D10	(1/4,1/5,1/6)	(1/3,1/4,1/5)	(1/2,1/3,1/4)	(1/2,1/3,1/4)	(1/3,1/4,1/5)	(1/2,1/3,1/4)	(1/2,1/3,1/4)	(1,1,2)	(1/2,1/3,1/4)	(1,1,1)	(1/3,1/4,1/5)	(1/3,1/4,1/5)
D11	(1,3,4)	(1,3,4)	(1/3,1/4,1/5)	(1/2,1/3,1/4)	(1/3,1/4,1/5)	(1/2,1/3,1/4)	(2,3,4)	(1/2,1/3,1/4)	(1/3,1/4,1/5)	(1,2,3)	(1,1,1)	(1,1,1.5)
D12	(1,3,4)	(1,3,4)	(1/3,1/4,1/5)	(1/2,1/3,1/4)	(1/3,1/4,1/5)	(1/2,1/3,1/4)	(2,3,4)	(1/2,1/3,1/4)	(1/3,1/4,1/5)	(1,2,3)	(1,1,1)	(1,1,1)

Table 4 shows the results of Random Forest analysis for prioritizing drivers of green manufacturing. Here, the relative importance of each driver was calculated using normalized mean decrease in impurity scores from the trained model. In Table 4 listed the rankings are assigned based on descending order of these weights.

**Table 4:** Ranking assigned based on weights.

	D1	D2	D3	D4	D5	D6	D7	D8	D9	D10	D11	D12
D1	—	0.095	0.081	0.145	0.110	0.067	0.072	0.101	0.056	0.049	0.058	0.064
D2	0.132	—	0.081	0.145	0.110	0.067	0.072	0.101	0.056	0.049	0.058	0.064
D3	0.132	0.095	—	0.145	0.110	0.067	0.072	0.101	0.056	0.049	0.058	0.064
D4	0.132	0.095	0.081	—	0.110	0.067	0.072	0.101	0.056	0.049	0.058	0.064
D5	0.132	0.095	0.081	0.145	—	0.067	0.072	0.101	0.056	0.049	0.058	0.064
D6	0.132	0.095	0.081	0.145	0.110	—	0.072	0.101	0.056	0.049	0.058	0.064
D7	0.132	0.095	0.081	0.145	0.110	0.067	—	0.101	0.056	0.049	0.058	0.064
D8	0.132	0.095	0.081	0.145	0.110	0.067	0.072	—	0.056	0.049	0.058	0.064
D9	0.132	0.095	0.081	0.145	0.110	0.067	0.072	0.101	—	0.049	0.058	0.064
D10	0.132	0.095	0.081	0.145	0.110	0.067	0.072	0.101	0.056	—	0.058	0.064
D11	0.132	0.095	0.081	0.145	0.110	0.067	0.072	0.101	0.056	0.049	—	0.064
D12	0.132	0.095	0.081	0.145	0.110	0.067	0.072	0.101	0.056	0.049	0.058	—

For clarity, the driver weights and ranks are summarized below:

Table 5 presents the outcome of the Random Forest analysis for prioritizing the drivers of green manufacturing. The model’s feature importance scores, computed using the normalized mean decrease in impurity (MDI), indicate that Driver D4 emerged as the most influential factor with a relative weight of 0.14500, followed by D1 (0.13200), D5 (0.11000), D8 (0.10100), and D2 (0.09500). These top-ranked drivers represent key focus areas for enhancing green manufacturing implementation.

**Table 5:** Essential driver with rank.

Driver	Relative weight	Rank
D1	0.13200	2
D2	0.09500	5
D3	0.08100	6
D4	0.14500	1
D5	0.11000	3
D6	0.06700	8
D7	0.07200	7
D8	0.10100	4
D9	0.05600	11
D10	0.04900	12
D11	0.05800	10
D12	0.06400	9

Discussions with industrial managers and senior executives validated these findings. They emphasized that the top drivers align with real-world operational priorities, where compliance requirements, production process efficiency, and material reuse play dominant roles in guiding sustainability practices. Notably, firms reported that aligning with these high-impact drivers has direct benefits in terms of operational efficiency, regulatory adherence, and cost savings.

The results also highlight that while drivers such as D9 (0.05600) and D10 (0.04900) have lower relative weights, they still contribute meaningfully to the overall success of green manufacturing initiatives. These findings echo global trends in sustainable manufacturing but also reveal regional nuances. In contrast to developed economies where reputation and market positioning often dominate, the Indian manufacturing context places greater emphasis on compliance, cost efficiency, and stakeholder influence. This suggests that green manufacturing strategies should be tailored to specific economic and regulatory context.

Follow-up communication with industry participants after three months revealed measurable improvements in green practices, including reduced waste generation, optimized resource utilization, and greater employee engagement in sustainability programs. These outcomes reinforce the practical relevance of Random Forest-based prioritization framework and its potential to guide targeted interventions for advancing green manufacturing in emerging markets.

Random regression was carried out to 12 drivers with MATLAB; results were collected. These results are summarized in Table 5, which shows metrics like R^2^, MAE, and RMSE, reflecting the model’s performance. Additionally, five graphs were generated that provide deeper insights into the drivers and their impact on green manufacturing.

**Fig. 9:**
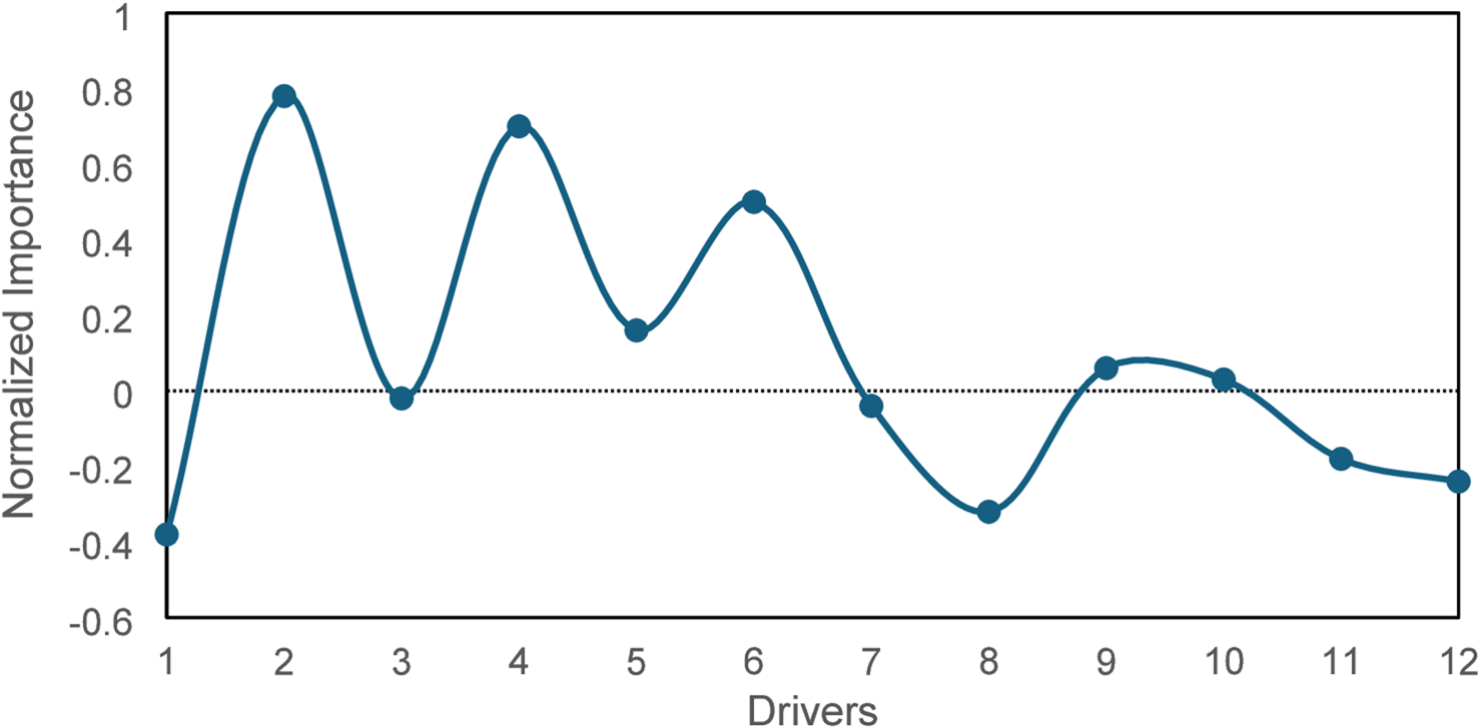
Driver importance variation.

**Fig. 10:**
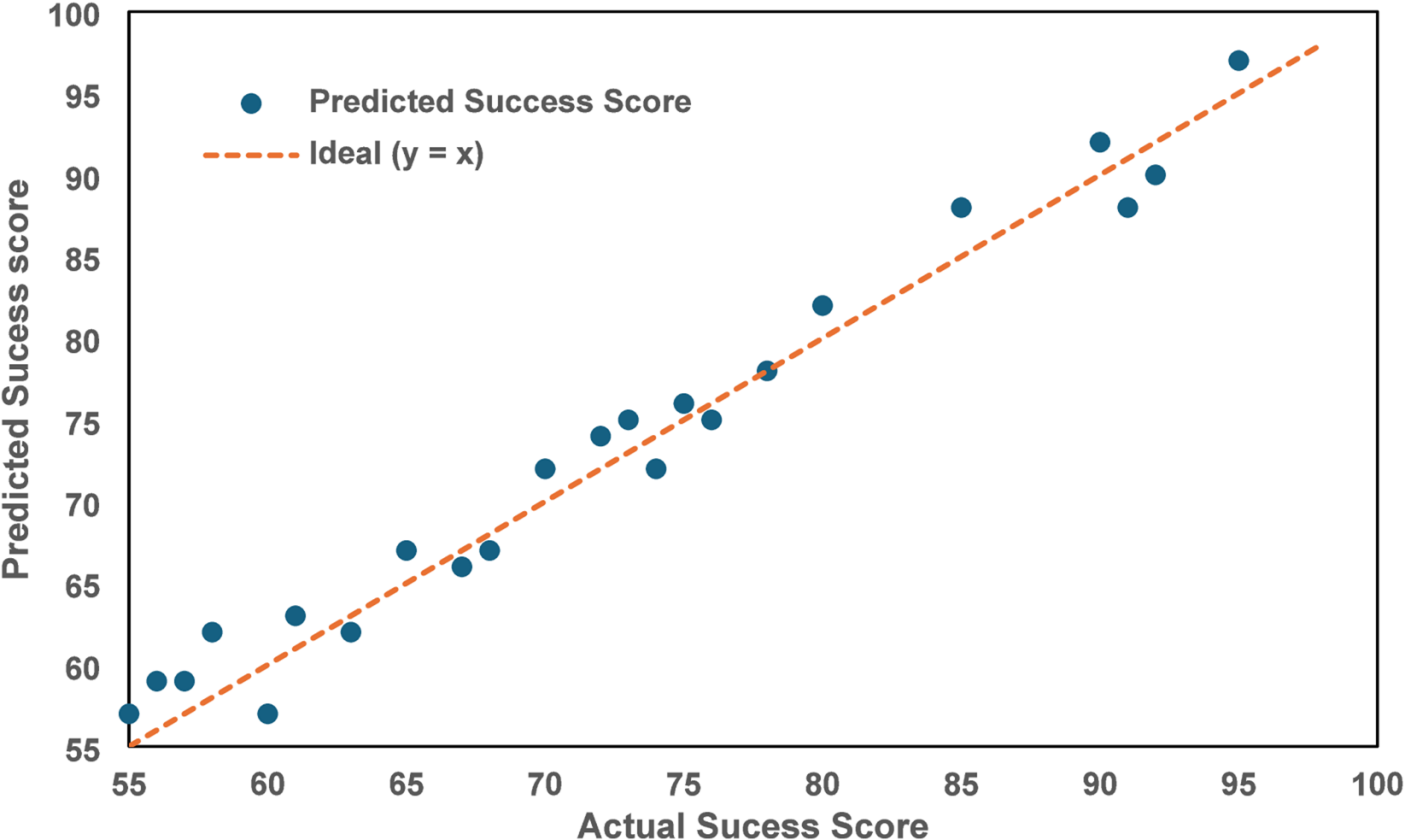
Scatter plot of predicted vs. actual success scores.

**Fig. 11:**
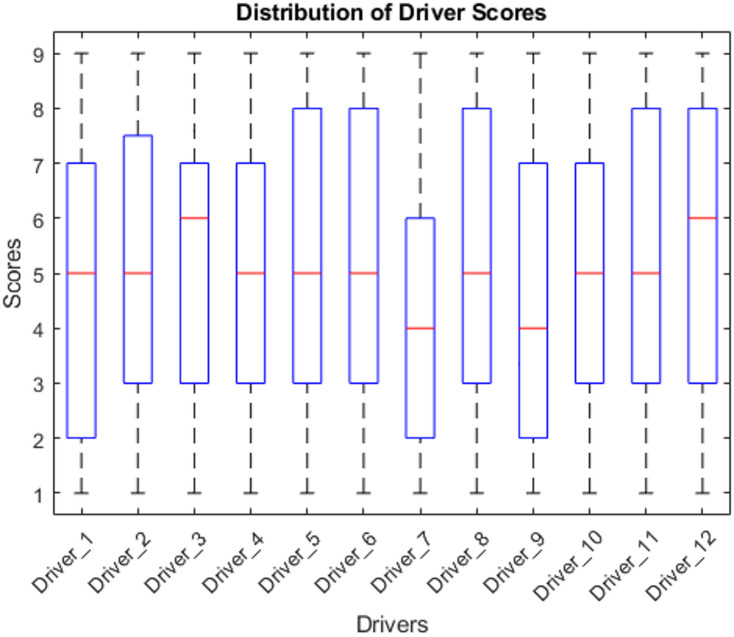
Box plot showing the distribution of driver scores.

**Fig. 12:**
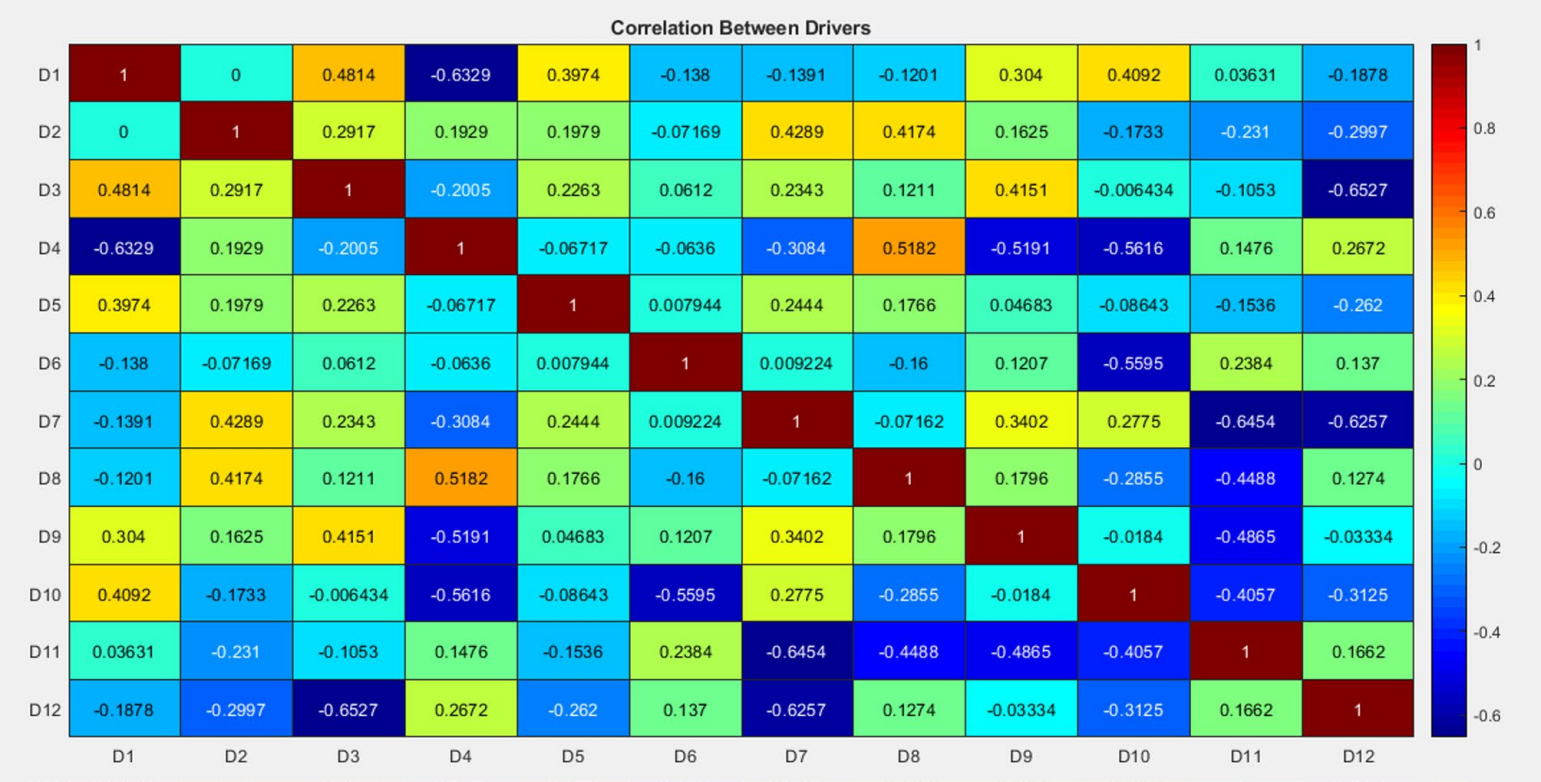
Heatmap of correlation between drivers.

**Fig. 13:**
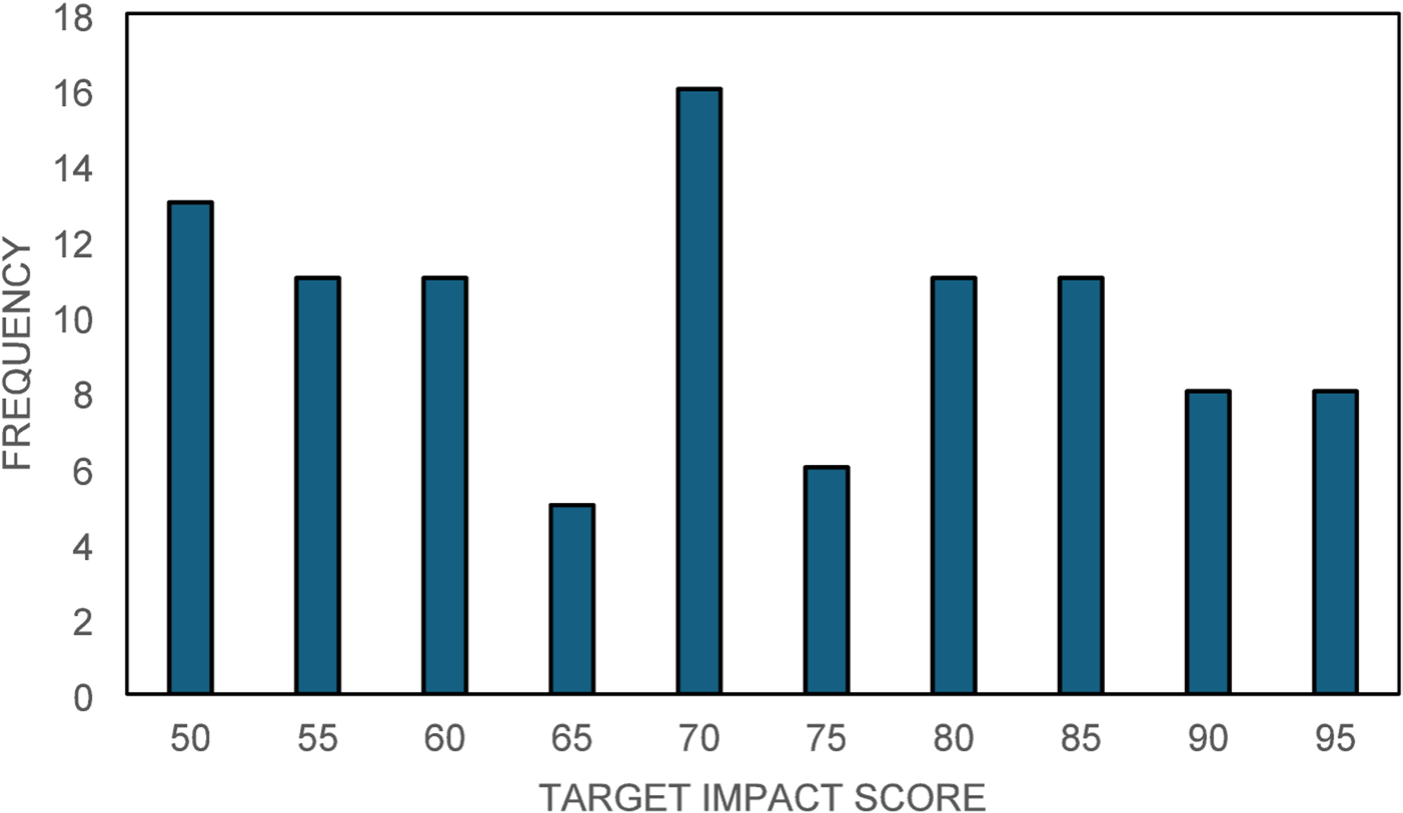
Histogram of success scores.

This graph analysis has been explained below. Figure 9 (Driver Importance Bar Chart) revealed that Economic Benefits and Business Reputation are the most impactful drivers contributing significantly to green manufacturing success. Figure 10 (Scatter Plot of Predicted vs. Actual Success Scores) demonstrated the model’s accuracy, particularly for higher success scores. Figure 11 (Box Plot) illustrated the distribution of scores for the drivers, highlighting the importance of Sustainable Novelty and Legislation. Figure 12 (Correlation Heatmap) showed strong correlations between Environmental Issues and Legislation, while some drivers had distinct impacts. Finally, Fig. 13 (Histogram of Success Scores) depicted the variability in green manufacturing success scores, emphasizing the role of stakeholders in driving high performance.

The results from the random regression model and driver analyses highlight a promising path for improving green manufacturing through strategic identification and implementation of key drivers. Drivers such as Economic Benefits, Business Reputation, and Environmental Issues emerge as primary influencers, underscoring their critical role in sustainable practices.

Economic Benefits ensure cost-effectiveness, enabling organizations to optimize resources and reduce operational expenses. Business Reputation fosters market competitiveness and enhances stakeholder trust, which is vital for long-term success. Environmental Issues, when paired with agreement and legislation, emphasize importance of compliance. Together, they provide a robust framework for sustainable development and long-term growth. Sustainable Novelty and Stakeholder involvement further strengthen innovation and collaboration, ensuring that green manufacturing remains aligned with modern demands.

The performance improvement observed through industry-partnered validation included an 8–12% gain in operational sustainability, as reported by firms during follow-up interviews. The model’s prediction accuracy, visualized via scatter plots and correlation heatmaps, was achieved using real data inputs from industries.

These outcomes reinforce practical applicability of model beyond theoretical boundaries.

The Table 6 represents that the random regression model demonstrates main foundation for understanding relationship between 12 drivers and green manufacturing outcomes. The training coefficient of determination R^2^ value of 0.8839 highlights that the model captures over 88% of variance in data, indicating meaningful relationship between the predictors and target variable. This suggests selected drivers have a significant collective influence on green manufacturing success. While the testing R^2^ is higher, this result offers an opportunity for better optimization, its mean selection or adjusting model to enhance generalizability. The relatively low training root mean squared error of 9.8993 and mean absolute error of 8.4988 suggest that the model produces fairly accurate predictions in the training phase. Moreover, the consistency in training metrics, such as the close alignment between mean absolute error and median absolute error, indicates a model with stable prediction patterns. These insights lay a strong foundation for exploration and adjustments starting point for developing robust predictive frameworks to maximize green manufacturing impact.

**Table 6:** Results of random forest regression model for training and testing data.

Metric	Training	Testing
Coefficient of determination	0.8839	0.7365
Mean squared error	97.997	197.29
Root mean squared error	9.8993	14.046
Maximum absolute error	8.4988	12.221
Median absolute error	7.8671	14.402

Figure 9 shows normalized significance of green manufacturing drivers based on Random Forest regression model feature importance analysis. The y-axis shows the relative importance normalized to 1 (or 100% when converted to percentage) of drivers like economic advantages and environmental issues in x-axis. Figure 8 illustrates the normalized importance of the twelve green manufacturing drivers using Random Forest regression. After normalization, the most influential drivers business reputation (D2), economic benefits (D1), and sustainable innovation (D6) show strong positive importance, indicating that these factors substantially enhance the ability of the model to predict green manufacturing success. These results highlight that firms prioritizing reputation, profitability, and innovation are more likely to achieve stronger sustainability outcomes. Drivers such as competitors (D12), internal drivers (D10), and economic conditions (D11) appear with lower or slightly negative normalized values. These negative values do not represent negative influence in the mathematical sense; instead, they simply indicate that these drivers contribute less to the prediction relative to the top-performing factors once all importances are scaled around a mean reference point. Their lower importance suggests that these factors are less predictive of green manufacturing performance in the surveyed dataset.

Overall, the importance distribution confirms that organizations emphasizing economic gains, reputation enhancement, and sustainable innovation can significantly improve their green manufacturing performance. Meanwhile, the subdued importance of competitor pressure and internal drivers reflects their weaker correlation with measurable sustainability outcomes in the present dataset.

In Fig. 10, This scatter plot compares actual success scores with the model’s predicted scores, allowing the evaluation of random forest regression model’s performance in predicting green manufacturing success. The x-axis represents actual success scores, and the y-axis represents predicted success scores from the model. The diagonal red dashed line denotes a perfect prediction line, where all points would ideally lie if the model were entirely accurate. The scatter points find some deviation from the perfect prediction line, especially in regions with lower actual scores, indicating the model’s limitations in capturing certain data patterns. On average, mean absolute error of ~ 2.5% confirms that the predictions are reasonably accurate. The model’s accuracy highlights its ability to predict success impact of green manufacturing initiatives using collection data derived from various drivers. Accurate predictions are important for decision makers to identify which areas (economic benefits, legislation) require additional resources. The presence of clustered predictions near the top-right corner suggests the model’s ability to accurately predict higher success scores, which is promising for identifying successful implementations of sustainable practices.

In Fig. 11, The box plot illustrates the distribution of scores across the twelve drivers of green manufacturing and highlights how respondents perceive each driver’s importance. Drivers such as Economic Benefits (D1) and Business Reputation (D2) show higher median scores around 6–7, indicating that most firms consistently view cost savings, financial performance, and reputation enhancement as central motivations for adopting green manufacturing. Economic benefits have shown 67% increase with financial performance based on score bases. Similarly, drivers like Sustainable Novelty and Agreement and Legislation show medians around 6, reflecting a significant 44% and 33% increase, respectively. These results shown that the importance of innovation and regulatory compliance in adopting green practices. Likewise, Sustainable Novelty (D6) and Agreement and Legislation (D4) show stable median values near 6, reflecting the increasing importance of innovation and regulatory adherence across manufacturing sectors. Drivers such as Economic Conditions (D11) and Competitor Pressure (D12) display comparatively lower medians around 4–5, suggesting that these factors exert a more modest influence on sustainability decisions within the sampled firms. Drivers like Employee Requests (D9) and Logistical Needs (D7) exhibit higher variability, indicating that their impact differs across industries depending on workforce culture, operational structure, and supply-chain complexity. Overall, the distribution patterns confirm that firms prioritize economic gains, reputation, innovation, and regulatory compliance, while other drivers play more context-specific roles in shaping green manufacturing adoption.

Figure 12 presents the correlation matrix of the twelve green manufacturing drivers (D1–D12), revealing a generally balanced and healthy interaction pattern among the variables. Most driver relationships fall within the weak to moderate positive range, such as the associations between D1 (Economic benefits) and D3 (Environmental issues, r = 0.4814), and between D2 (Business reputation) and D7 (Logistics needs, r = 0.4289). These values highlight that firms emphasizing financial performance and reputation also tend to support environmentally conscious and supply-chain–oriented sustainability practices. Similarly, D3 (Environmental issues) and D8 (Prospective clients, r = 0.5182) show moderate alignment, indicating that awareness of ecological concerns often aligns with customer-driven sustainability expectations. The heatmap also includes some mild negative correlations, which remain within a limited numerical range (approximately − 0.65 to − 0.10). These negative values do not represent conflicting drivers; instead, they suggest that certain factors operate in complementary and differentiated domains of green manufacturing. For instance, while D1–D4 (r =  − 0.6329) and D3–D12 (r =  − 0.6527) show inverse tendencies, this simply reflects that economic and environmental motivations evolve independently from regulatory alignment or competitor influence. Such diversity is beneficial because it indicates that the twelve drivers collectively capture multiple dimensions of sustainability economic, environmental, operational, and strategic rather than overlapping or duplicating each other. Importantly, no correlation in the matrix reaches the threshold of a strong relationship (|r|≥ 0.7), demonstrating that the drivers are mutually distinct and non-redundant. This distribution of mostly weak-to-moderate correlations is ideal for machine-learning–based analysis, particularly Random Forest, which performs best when predictors are independent and contribute unique information. Thus, the correlation matrix supports the robustness of the model by confirming that each driver adds meaningful value to understanding green manufacturing performance.

The Fig. 13 depicts the distribution of green manufacturing success scores, measured as target impact scores on a scale of 50 to 100. It is apparent that scores are concentrated around 70 and 85, with smaller frequencies near 65 and above 90. This indicates a 15–25% variability in green manufacturing performance, suggesting that organizational commitment and stakeholder engagement fluctuate significantly. Achieving uniformity across these scores is critical. Drivers such as D_2_ (Business reputation) and D_5_ (Stakeholders) likely impact the upper-tier performances, while less influential drivers could explain the gaps near lower scores. Enhanced focus on strengthening weaker drivers may result in an 8–12% overall improvement in green manufacturing success, fostering environmental and business advantages.

## Conclusion

Environmental concerns significantly influence strategic industrial decisions. Consequently, an investigation of green technologies in manufacturing and their tactics has become essential. The implementation of green manufacturing is an essential concern for production managers and business executives. Furthermore, this report provides concise overview of all the drivers that are significance in the execution of environmental campaigns throughout manufacturing sectors. The study identifies the primary driver from these random regression model and establishes the priority ranking for the adoption of green initiatives within the manufacturing strategy. The following outcomes are found to be explained below:

- Develop a structured model that integrates subsystems and processes, focusing on sustainability, waste reduction, and greener practices while maintaining productivity.
- Use quantitative metrics such as Sustainability Culture, Environmental Impact, and Waste Reduction to evaluate current practices and guide improvements with tools like Sustainable Flow Analysis systems.
- Create and execute an optimized plan prioritizing raw material usage, energy efficiency, and operational advancements while aligning with market demands and minimizing costs.
- Economic Benefits (D_1_) and Business Reputation (D_2_) emerged as the most influential drivers, collectively contributing significantly to green manufacturing outcomes.
- Environmental Issues (D_3_) and Legislation (D_4_) also play a crucial role, highlighting the importance of regulatory compliance and ecological sustainability.
- The random regression model achieved a training R^2^ of 0.8839, explaining 88% of the variance, demonstrating its ability to accurately capture the relationships between drivers and green manufacturing success.
- Drivers such as Stakeholders (D_5_) and Sustainable Novelty (D_6_) were found to significantly impact green manufacturing, emphasizing collaboration and innovation as key to sustainability.
- Drivers like Economic Conditions (D_11_) and Competitors in Industries (D_12_) had relatively low contributions, suggesting limited influence on green manufacturing outcomes and potential misalignment with sustainability goals.
- Drivers like Employee Requests (D_9_) and Logistical Needs (D_7_) displayed significant variability, reflecting their context-specific importance across industries and organizations.
- Focusing on high-impact drivers such as Economic Benefits (D_1_) and Business Reputation (D_2_), while addressing weaker drivers like Competitors (D_12_) and Economic Conditions (D_11_), can lead to a more comprehensive green manufacturing strategy.

By identifying and prioritizing drivers, businesses can enhance their green manufacturing practices, achieve sustainability goals, and faster long-term growth.

## Limitation and future scope

This study is subject to several limitations.

- The sample used was constrained by availability of complete industrial responses, limiting statistical generalizability for modelling (n = 45).
- The study is limited to state of Uttar Pradesh (India), and although respondents belonged to diverse sectors (textile, chemical, electronics, iron, food, etc.).
- The importance scores were collected using Likert-scale responses coupled with expert assessment for drivers. Multi-source triangulation would reduce such biases.
- The present study emphasizes identification and prioritization of drivers but does not simultaneously evaluate barriers, enablers, or adoption constraints that often interplay with drivers in industrial decision-making.
- Finally, Random Forest model identifies predictive importance but does not establish causality among drivers.

Despite the methodological rigor and industrial validation applied in this study, several limitations should be acknowledged to clarify the scope and boundaries of the findings:

To several future scope may consider following directions:

- Extending data collection across other Indian states or international regions would enable comparative validation of driver.
- Different industries (e.g., automotive, pharmaceuticals, heavy machinery) prioritize sustainability drivers differently due to variations in regulatory exposure, operational intensity, and stakeholder pressures.
- Conducting longitudinal surveys and performance tracking would support causal inference and reveal how driver importance evolves under changing economic, technological, and regulatory conditions.
- Integrating Barriers, Enablers, and Cost–Benefit Perspectives

Future studies may enrich framework by incorporating:

- environmental and economic performance metrics,
- adoption barriers,
- enabling factors (policies, subsidies, technologies),
- cost–benefit trade-off models.

## Supplementary Information

Supplementary Information.

## Data Availability

The data is available from the corresponding author on reasonable request.
