# Supplementary material for: Identifying and ranking drivers of green manufacturing for sustainable industrial practices
Source: Sci Rep. 2026 Apr 8;16:16647. doi: 10.1038/s41598-026-44849-y (PMC13219587; doi:10.1038/s41598-026-44849-y)
Supplement: Supplementary file 1 — Supplementary Information. [file 41598_2026_44849_MOESM1_ESM.docx]

## ****Annexure-I****

## ****Industry Survey Questionnaire for Industrial Respondents****

(For Green Manufacturing Driver Assessment)

**Purpose:**
This questionnaire is designed to collect expert insights from manufacturing professionals regarding the importance and practical relevance of twelve key drivers influencing the adoption of Green Manufacturing (GM) practices in Indian industries. Your responses will be used exclusively for academic research.

**Instructions:**

- Please rate each driver on a **1–9 Likert scale**, where:
  **1 = Not Important**,
  **5 = Moderately Important**,
  **9 = Extremely Important**.

## ****Section A: Respondent and Organization Profile****

1. **Name (Optional):** _________________________
2. **Designation:** _____________________________
3. **Department:** _____________________________
4. **Industry Type (Automobile/Steel/Chemical/Textile/Others):** ________
5. **Years of Experience in Manufacturing:**

- 0–5 years
- 5–10 years
- 10–20 years
- 20 years

1. **Organization Size:**

- Small
- Medium
- Large

1. **ISO Certifications:**

- ISO 9001
- ISO 14001
- ISO 50001
- None

## ****Section B: Rating of Green Manufacturing Drivers****

Please rate the importance of the following 12 drivers.

### ****D1. Economic Benefits****

Extent to which GM improves cost savings, energy optimization, waste reduction, and long-term financial performance.
**Rating (1–9):**

### ****D2. Government Regulations & Policies****

Influence of environmental laws, pollution control norms, and compliance requirements on GM adoption.
**Rating (1–9):**

### ****D3. Environmental Sustainability Pressure****

Firm’s need to reduce emissions, conserve resources, and meet environmental protection standards.
**Rating (1–9):**

### ****D4. Customer Demand & Market Competitiveness****

Impact of customer expectations for eco-friendly products and pressure to remain competitive.
**Rating (1–9):**

### ****D5. Technological Advancement & Automation****

Availability of modern technologies (IoT, AI, robotics, energy-efficient systems) that support GM.
**Rating (1–9):**

### ****D6. Top Management Commitment****

Strategic support, policy decisions, and resource allocation by executive leadership for GM.
**Rating (1–9):**

### ****D7. Employee Awareness & Training****

Skill development, awareness programs, and involvement of employees in GM initiatives.
**Rating (1–9):**

### ****D8. Supplier & Stakeholder Pressure****

Requirements from suppliers, vendors, and global partners to adopt green practices.
**Rating (1–9):**

### ****D9. Corporate Social Responsibility (CSR)****

Role of CSR initiatives in motivating industries toward sustainable and green processes.
**Rating (1–9):**

### ****D10. Resource Availability****

Availability of renewable resources, cleaner materials, and sustainable inputs.
**Rating (1–9):**

### ****D11. Waste Management & Recycling Practices****

Effectiveness of waste handling, material recovery, and recycling systems.
**Rating (1–9):**

### ****D12. Energy Efficiency Initiatives****

Adoption of energy-saving technologies, renewable energy systems, and efficiency programs.
**Rating (1–9):**

## ****Section C: Additional Insights****

1. **What are the major barriers your organization faces in implementing Green Manufacturing?**
2. **What strategies could improve green manufacturing adoption in Indian industries?**
3. **Any suggestions for enhancing sustainability performance in your organization?**

**Form Title**:
"Green Manufacturing Drivers Questionnaire"

**Form Description**:
"This form is designed to collect data for evaluating the drivers of green manufacturing in industries. The information provided will help prioritize strategies to enhance sustainability in manufacturing processes. Please answer all questions carefully."

**Form Sections and Questions**

**Section 1: Company and Respondent Details**

1. **Company Name** *(Short Answer)*
2. **Executive Name** *(Short Answer)*
3. **Email ID** *(Short Answer)*
4. **Contact Number** *(Short Answer)*
5. **Company Field/Industry** *(Dropdown)*:
   - Iron
   - Automobile
   - Electronics
   - Textile
   - Chemical/Paint
   - Food
   - Paper
   - Others
6. **Annual Turnover** *(Short Answer)*
7. **Number of Employees** *(Short Answer)*

**Section 2: Objective Questions (50 Questions)**

Each question is tick-based, with multiple-choice or checkbox options. Questions are grouped by driver objectives:

**Objective 1: Economic Benefits**

1. Is cost reduction a primary motivation for adopting green manufacturing?
   - Always
   - Sometimes
   - Rarely
   - Never
2. Does your company measure financial gains from green manufacturing?
   - Always
   - Sometimes
   - Rarely
   - Never
3. Are energy-saving techniques utilized in production?
   - Always
   - Sometimes
   - Rarely
   - Never
4. Is resource optimization directly linked to financial performance in your company?
   - Strongly Agree
   - Agree
   - Neutral
   - Disagree

**Objective 2: Business Reputation**

1. Do you believe green manufacturing improves customer trust?
   - Always
   - Sometimes
   - Rarely
   - Never
2. Has adopting green practices enhanced your company’s brand image?
   - Always
   - Sometimes
   - Rarely
   - Never
3. Does your company promote green manufacturing in its marketing campaigns?
   - Always
   - Sometimes
   - Rarely
   - Never
4. Do customers inquire about the sustainability of your products?
   - Always
   - Sometimes
   - Rarely
   - Never

**Objective 3: Environmental Issues**

1. Has your company implemented waste reduction programs?
   - Always
   - Sometimes
   - Rarely
   - Never
2. Is minimizing your carbon footprint a key focus of operations?
   - Always
   - Sometimes
   - Rarely
   - Never
3. Does your company actively monitor environmental compliance standards?
   - Always
   - Sometimes
   - Rarely
   - Never
4. Are environmental audits conducted regularly?
   - Always
   - Sometimes
   - Rarely
   - Never

**Objective 4: Agreement and Legislation**

1. Is your company certified for ISO 14001 or other environmental standards?
   - Always
   - Sometimes
   - Rarely
   - Never
2. How often do you update policies to align with government regulations?
   - Always
   - Sometimes
   - Rarely
   - Never
3. Are penalties for non-compliance a significant concern for your company?
   - Always
   - Sometimes
   - Rarely
   - Never
4. Is aligning with green manufacturing regulations considered a strategic advantage?
   - Always
   - Sometimes
   - Rarely
   - Never

**Objective 5: Stakeholders**

1. Do stakeholders actively encourage green practices in your company?
   - Always
   - Sometimes
   - Rarely
   - Never
2. Is green manufacturing an expectation from your investors?
   - Always
   - Sometimes
   - Rarely
   - Never
3. Are employees motivated to participate in green initiatives?
   - Always
   - Sometimes
   - Rarely
   - Never
4. How often do stakeholders request sustainability updates?
   - Always
   - Sometimes
   - Rarely
   - Never

**Objective 6: Sustainable Novelty**

1. Has your company adopted innovative green technologies recently?
   - Always
   - Sometimes
   - Rarely
   - Never
2. Are product designs influenced by eco-friendly considerations?
   - Always
   - Sometimes
   - Rarely
   - Never
3. Do you prioritize research into sustainable solutions?
   - Always
   - Sometimes
   - Rarely
   - Never
4. Are innovation-driven green practices more costly to implement?
   - Always
   - Sometimes
   - Rarely
   - Never

**Objective 7: Logistical Needs**

1. Does your company use recyclable packaging materials?
   - Always
   - Sometimes
   - Rarely
   - Never
2. Are reverse logistics (recycling/reuse) part of your supply chain?
   - Always
   - Sometimes
   - Rarely
   - Never
3. How often are materials reused within your supply chain?
   - Always
   - Sometimes
   - Rarely
   - Never
4. Is supply chain optimization considered a green initiative?
   - Always
   - Sometimes
   - Rarely
   - Never

**Objective 8: Prospective Clients**

1. Do clients prioritize sustainability in their purchase decisions?
   - Always
   - Sometimes
   - Rarely
   - Never
2. Have you gained new clients by adopting green manufacturing?
   - Always
   - Sometimes
   - Rarely
   - Never
3. Do customer demands drive your green manufacturing practices?
   - Always
   - Sometimes
   - Rarely
   - Never
4. Are clients willing to pay more for sustainable products?
   - Always
   - Sometimes
   - Rarely
   - Never

**Objective 9: Employee Requests**

1. Do employees prefer working for environmentally responsible companies?
   - Always
   - Sometimes
   - Rarely
   - Never
2. Has employee satisfaction improved due to green initiatives?
   - Always
   - Sometimes
   - Rarely
   - Never
3. Are employees trained in sustainable manufacturing practices?
   - Always
   - Sometimes
   - Rarely
   - Never
4. Is employee feedback considered for green manufacturing decisions?
   - Always
   - Sometimes
   - Rarely
   - Never

**Objective 10: Internal Drivers**

1. Do internal policies emphasize green manufacturing?
   - Always
   - Sometimes
   - Rarely
   - Never
2. Is there a dedicated green manufacturing team?
   - Always
   - Sometimes
   - Rarely
   - Never
3. Are green initiatives linked to employee performance metrics?
   - Always
   - Sometimes
   - Rarely
   - Never
4. Are internal resources sufficient to support green manufacturing?
   - Always
   - Sometimes
   - Rarely
   - Never

**Objective 11: Economic Conditions**

1. Does your company rely on external funding for green initiatives?
   - Always
   - Sometimes
   - Rarely
   - Never
2. Are economic downturns a barrier to adopting green practices?
   - Always
   - Sometimes
   - Rarely
   - Never
3. Does economic stability influence your investment in green manufacturing?
   - Always
   - Sometimes
   - Rarely
   - Never
4. Are cost-benefit analyses performed before implementing green practices?
   - Always
   - Sometimes
   - Rarely
   - Never

**Objective 12: Competitors**

1. Do competitors influence your decision to adopt green manufacturing?
   - Always
   - Sometimes
   - Rarely
   - Never
2. Are competitors adopting similar sustainability measures?
   - Always
   - Sometimes
   - Rarely
   - Never
3. Is competition driving innovation in green practices?
   - Always
   - Sometimes
   - Rarely
   - Never
4. Do competitors' strategies influence your green manufacturing timelines?
   - Always
   - Sometimes
   - Rarely
   - Never

**Final Section: Overall Opinion**

1. Do you believe green manufacturing is critical for long-term success?
   - Always
   - Sometimes
   - Rarely
   - Never
2. Would you recommend green manufacturing to other industries?
   - Always
   - Sometimes
   - Rarely
   - Never

**Manual Counting**

For a small sample size, you can manually count responses:

1. **Create a table** for each question with response categories as columns (e.g., Never, Rarely, Sometimes, Always).
2. **Record counts** for each response.

**Example Table for Question 1:**

| **Response** | **Count** |
| --- | --- |
| Never | 10 |
| Rarely | 15 |
| Sometimes | 20 |
| Always | 5 |

**Using a Spreadsheet (Excel/Google Sheets)**

1. **Input Data:**
   - Each row is a participant's response.
   - Each column represents a question.

| **Participant** | **Question 1** | **Question 2** | **Question 3** |
| --- | --- | --- | --- |
| 1 | Sometimes | Always | Rarely |
| 2 | Rarely | Never | Always |
| 3 | Always | Sometimes | Sometimes |
